# Sunlight Modulates Fruit Metabolic Profile and Shapes the Spatial Pattern of Compound Accumulation within the Grape Cluster

**DOI:** 10.3389/fpls.2017.00070

**Published:** 2017-02-01

**Authors:** Noam Reshef, Natasha Walbaum, Nurit Agam, Aaron Fait

**Affiliations:** French Associates Institute for Agriculture and Biotechnology of Drylands, The Jacob Blaustein Institutes for Desert Research, Ben-Gurion University of the NegevSede Boqer, Israel

**Keywords:** solar irradiance, microclimate, spatial heterogeneity, grape composition, phenylpropanoids, metabolite profiling, climate change, shading

## Abstract

Vineyards are characterized by their large spatial variability of solar irradiance (SI) and temperature, known to effectively modulate grape metabolism. To explore the role of sunlight in shaping fruit composition and cluster uniformity, we studied the spatial pattern of incoming irradiance, fruit temperature and metabolic profile within individual grape clusters under three levels of sunlight exposure. The experiment was conducted in a vineyard of Cabernet Sauvignon cv. located in the Negev Highlands, Israel, where excess SI and midday temperatures are known to degrade grape quality. Filtering SI lowered the surface temperature of exposed fruits and increased the uniformity of irradiance and temperature in the cluster zone. SI affected the overall levels and patterns of accumulation of sugars, organic acids, amino acids and phenylpropanoids, across the grape cluster. Increased exposure to sunlight was associated with lower accumulation levels of malate, aspartate, and maleate but with higher levels of valine, leucine, and serine, in addition to the stress-related proline and GABA. Flavan-3-ols metabolites showed a negative response to SI, whereas flavonols were highly induced. The overall levels of anthocyanins decreased with increased sunlight exposure; however, a hierarchical cluster analysis revealed that the members of this family were grouped into three distinct accumulation patterns, with malvidin anthocyanins and cyanidin-glucoside showing contrasting trends. The flavonol-glucosides, quercetin and kaempferol, exhibited a logarithmic response to SI, leading to improved cluster uniformity under high-light conditions. Comparing the within-cluster variability of metabolite accumulation highlighted the stability of sugars, flavan-3-ols, and cinnamic acid metabolites to SI, in contrast to the plasticity of flavonols. A correlation-based network analysis revealed that extended exposure to SI modified metabolic coordination, increasing the number of negative correlations between metabolites in both pulp and skin. This integrated study of micrometeorology and metabolomics provided insights into the grape-cluster pattern of accumulation of 70 primary and secondary metabolites as a function of spatial variations in SI. Studying compound-specific responses against an extended gradient of quantified conditions improved our knowledge regarding the modulation of berry metabolism by SI, with the aim of using sunlight regulation to accurately modulate fruit composition in warm and arid/semi-arid regions.

## Introduction

Plasticity is the ability of an organism to adapt its phenotype in response to changes in the environment. For plants, it is an important adaptive strategy to cope with environmental variability (Svanback and Eklov, 2006) and facilitates the use of the same cultivar in a wide range of growing conditions (Dai et al., 2011). However, at the single-field scale, phenotypic plasticity may be regarded as a significant cause for within-field variation. Field systems, such as vineyards experience a large range of microclimate conditions manifested in significant spatial variation of SI and temperature (Oyarzun et al., 2007; Matese et al., 2014).

Grape and wine quality reflects the levels and composition of a large number of primary and secondary metabolites that shape the overall sensory experience of its derived product. Primary metabolites are largely accumulated in the pulp and include sugars, organic acids, and amino acids. Grape secondary metabolites predominantly include the phenylpropanoids, typically found in the berry skin, and comprise flavonoids, phenolic acids, stilbenes, and viniferins (Anesi et al., 2015). While sugars are largely imported to the fruit through the plant vascular tissues (Ollat et al., 2002), the overall levels and composition of organic acids, amino acids, and secondary metabolites at harvest are determined by the sum of complex metabolic processes occurring in the fruit during its lifetime (Conde et al., 2007; Deluc et al., 2007; Dai et al., 2013). Indeed, metabolic shifts caused by changes in climatic conditions in the immediate vicinity of the fruit (i.e., microclimate), such as light and temperature, are well-known to affect fruit composition (Jackson and Lombard, 1993; Downey et al., 2006).

Studies exploring the effect of microclimate on fruit primary metabolites largely agree that sunlight, and specifically an increase in the temperature load of the fruit, highly modulate malic acid levels, leading to a corresponding decrease in overall fruit acidity (Kliewer, 1965; Jackson and Lombard, 1993; Conde et al., 2007; Sweetman et al., 2014). However, results regarding the effect on the relatively stable tartaric acid are still controversial (Rienth et al., 2016; Young et al., 2016). Discrepancies also exist regarding the effect on amino acids, owing in part to the differential response of cultivated varieties. For example, filtering UV-B irradiance was found to increase the overall levels of amino acids in the juice of Riesling cv. (Schultz et al., 1998), but had no effect on the levels and composition of amino acids in Sauvignon Blanc cv. grapes at harvest (Gregan et al., 2012).

With regards to grape specialized metabolism, studies underline the flavonoids as a highly responsive group to light and temperature perturbations (Chalker-Scott, 1999; Winkel-Shirley, 2002). For instance, flavonol glucoside concentrations in the fruit positively correlated with increased SI on the cluster (Haselgrove et al., 2000; Spayd et al., 2002), and were found to be the most significant metabolites distinguishing the metabolic profile of shaded berries from that of exposed berries (Pereira et al., 2006). Fruit sunlight exposure was found to modulate the composition of anthocyanins, such as the proportion of acylated and coumarylated forms, the ratio between di-hydroxylated and tri-hydroxylated anthocyanins (Haselgrove et al., 2000; Spayd et al., 2002; Downey et al., 2004; Tarara et al., 2008; Chorti et al., 2010), and the proportion of ortho-di phenol anthocyanins (Rustioni et al., 2011). However, the overall accumulation of anthocyanins showed higher dependency on temperature conditions than on SI (Downey et al., 2006), with the effect of the interaction between incoming SI and fruit temperature on grape anthocyanin levels exhibiting a synergistic or antagonistic character, depending on the ranges of both factors (Tarara et al., 2008; Azuma et al., 2012).

While the overall environmental effect on grape has been investigated, few studies have addressed the within-cluster spatial profile of compound accumulation and variability. Pisciotta et al. (2013) characterized berry anthocyanin content considering its vertical position on the rachis and external and internal faces of the cluster (i.e., facing the inter-row or the canopy). However, no data regarding micrometeorological conditions were presented, hampering the interpretation of the results with respect to environmental conditions. Zhang et al. (2015) found greater polyphenol content in regions of the cluster characterized by lower berry surface temperature in Shiraz cv. More recently, by using model-generated microclimate data, Pieri et al. (2016) related the overall levels of berry flavonols, anthocyanins, and amino acids to cluster exposition and its impact on the estimated levels of SI and berry surface temperature (BST). Taken together, these works indicate that micro-scale environments play an active role in generating the spatial patterns of metabolite accumulation that affect fruit composition, as well as the uniformity of the crop. Optimizing crop uniformity is generally regarded as an important aspect in the overall improvement of wine quality (Keller, 2010), owing to its significant role in the composition of the harvested grape, and final wine (Kontoudakis et al., 2011; Liu et al., 2016). However, recent attempts to minimize field heterogeneity through the use of common vineyard management techniques, such as deficit irrigation and cluster thinning, were found inefficient (Calderon-Orellana et al., 2014). This stresses the importance of exploring the effectiveness of other potential techniques, including the modulation of cluster-zone microclimate, to form more homogeneous conditions. As a key factor determining fruit intercepted irradiance and temperature (Smart and Sinclair, 1976), practical interventions to ameliorate fruit microclimate generally involve the regulation of sunlight exposure. Practices, such as basal leaf removal, or shading, whether by the use of artificial nets or by the use of extended canopies, are routinely used in the industry. However, integrated studies encompassing a gradient of sunlight exposure, profiling of metabolic data, and a quantitative characterization of microclimate conditions are lacking. Hence, our understanding of how sunlight regulation influences the spatial heterogeneity of fruit microclimate and its effect on fruit metabolic processes and coordination is currently limited.

In this study, we performed a spatial characterization of micrometeorological conditions and metabolic profiles within individual grape clusters subjected to differing levels and directions of sunlight exposure. The extended range of SI intensities allowed us to explore the responses of a large number of primary and secondary (phenylpropanoids) metabolites to a range of microclimate conditions typically found in the field. This study's insights will aid in defining strategies for sunlight regulation aimed at improving fruit composition and uniformity in challenging environments.

## Materials and methods

### Site description

The experiment was conducted during the 2014 and 2015 growing seasons, in a vineyard located in the heart of the Negev Desert, Israel (30°36′55.22″N, 34°45′12.00″E, 800m altitude). This is an arid region with an average annual precipitation rate of 70mm (Israel Meteorological Service), which, during the growing season, is characterized by stable meteorological conditions including high SI and elevated midday temperatures (Supplementary Figure 1). The vineyard was planted in 2007 with *Vitis Vinifera* L. cv. Cabernet Sauvignon grafted on 140 Ruggeri rootstock, irrigated using a covered drip-irrigation system as is common in the region. Rows are orientated north-south with a 30° angle to the north-east\south-west, and are trained in vertical shoot positioning (VSP). Three experimental rows were selected, with one border row between every two experimental rows. In each row, three groups of nine adjacent vines were marked as field repetitions, and the basal leaves in the vicinity of the clusters (up to 30 cm above the cordon) were completely removed at the onset of veraison. Each field repetition was further assigned one of three shading treatments: fully exposed, i.e., no net (Exposed), 30% shading net (30% shaded) and 60% shading net (60% shaded), using UV-stabilized woven mesh shading nets for agriculture (Ginegar, Israel) with the percentage representing the PAR filtering capacity as published by the manufacturer. The nets were placed using thin metal support wires, one next to each vine, in a manner that created a shading tunnel with a diameter of about 80 cm around the cluster zone in order to facilitate wind flow and prevent an increase in relative humidity (Supplementary Figure 2). Shading was placed from the onset of veraison (the day of basal leaf removal) until harvest date. Treatments were repeated once on each row in a way that represented all locations along the row to minimize effects of spatial differences both between and within the rows.

### Meteorological measurements

During the 2015 growing season, a detailed study of cluster-scale micrometeorological conditions was performed. Incoming SI, air temperature, relative humidity (RH%), and wind speed and direction were continuously monitored in 15-min intervals from veraison to harvest by placing a multi-sensor (WS501-UMB, Lufft, Fellbach, Germany) connected to a data logger (CR200, Campbell Scientific, Utah, USA) 1m above the canopy, positioned in the field to allow for a maximum fetch in the direction of the prevailing winds (northwest). The distance between the sensor and the most distant experimental vine was approximately 90 m.

Cluster-zone air temperature and RH% were measured continuously every 15 min from veraison to harvest by placing sensors equipped with an internal data logger (Hobo ProV2, Onset, MA, USA) at a shaded spot in the immediate vicinity of the clusters. Two sensors were placed at each treatment site to verify repeatability.

The SI intensity on the clusters was measured simultaneously at four horizontal axes: parallel and perpendicular to the vine row, and vertically, to the sky (Supplementary Figure 3). This was done by constructing a box positioning all five pyranometers (LI200R, Li-Core, NE, USA) and connecting them to a single data logger (21X, Campbell Scientific, UT, USA). The sensors were stabilized and leveled on a tripod and placed at the cluster zone, with representative locations and distances from adjacent clusters verified to simulate cluster conditions. This system was continuously rotated every 3 days between field treatments and normalized to the SI measured above the canopy to allow for a comparison between measurements made on different dates. Measurements were done on the east side of the canopy for the shaded treatments and on both sides of the canopy for the exposed treatment.

Berry surface temperature (BST) was measured on 28 July (post-veraison) and 19 August (pre-harvest) 2015, during two diurnal field campaigns from 6:00 to 20:00 using an infrared camera (T640, FLIR systems, OR, USA). Every hour, photos were taken from two representative clusters on each treatment, from each side of the canopy, for a total of 12 clusters. Each cluster was divided into three faces, north- and south-facing, and facing the inter-row (east for east-located clusters and west for west-located clusters). Data analysis included a careful selection of berries located in the middle section of the cluster's vertical axis (i.e., middle height) in each photo and the exclusion of pixels representing non-grape background data.

### Berry sampling

**Figure 1:**
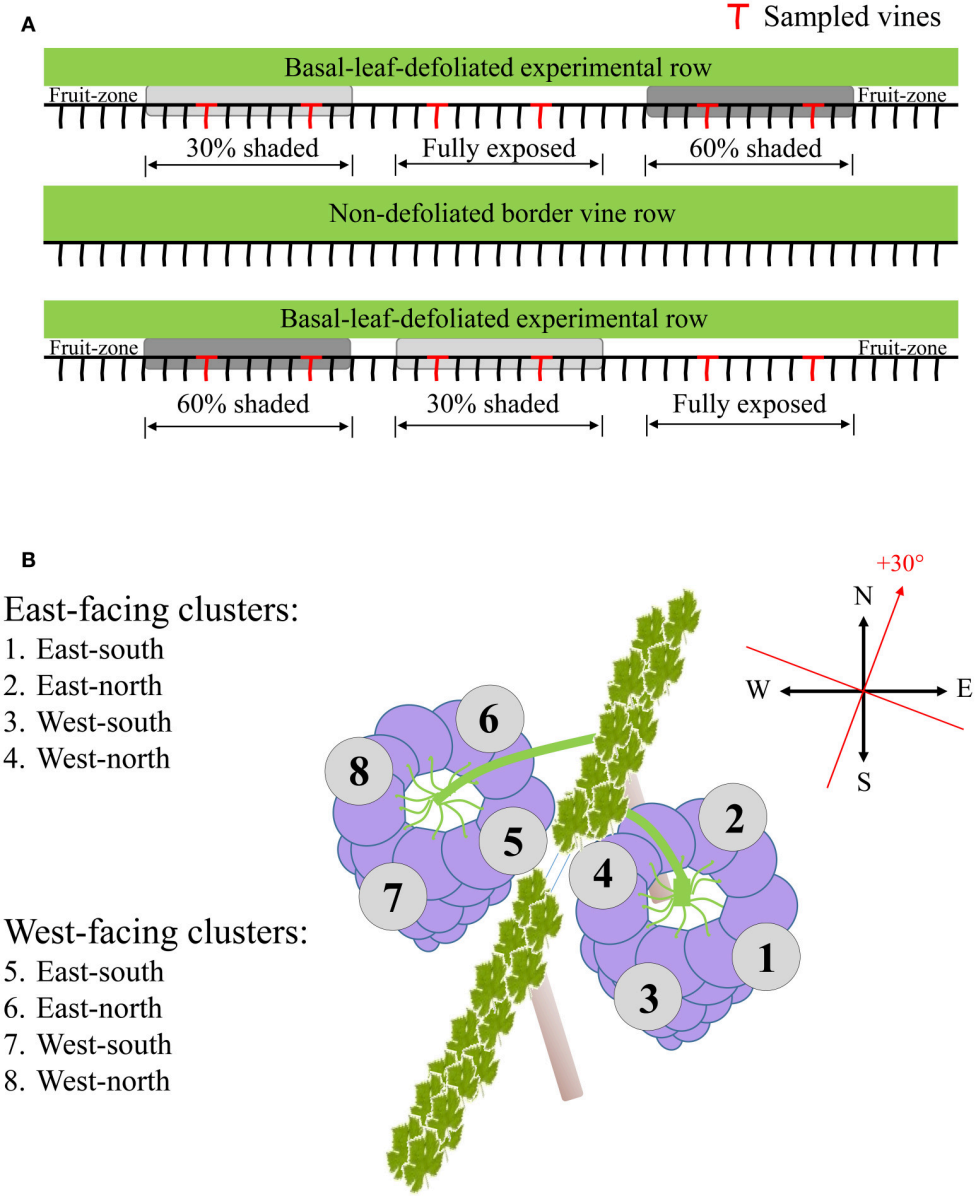
**Sampling layout illustrating the selection of experimental vines (A)**, and the dissection of clusters of both canopy sides to the four orientations **(B)**.

Sampling during both seasons was done several days prior to harvest date scheduled at 24°Brix, to represent final berry composition and the potential of accumulated spatial differences. Samples were taken at pre-dawn in order to prevent any differences between sunlit and shaded berries at the time of sampling. Four vines per shading treatment were sampled, located in two out of the three field repetitions. Selected vines for sampling were at least four vines apart (Figure 1A). In each vine, four to six exterior-located clusters (furthest from the trunk toward the inter-row) were selected from each canopy side, to avoid possible shading by the canopy apart from midday hours. Each cluster was dissected into four horizontal orientations (Figure 1B), and samples were taken from the middle height of each orientation. Berries from a single vine and canopy side, located on the same cluster plane, were pooled together and immediately frozen in liquid nitrogen. In the lab, skin, pulp and seeds were carefully separated while kept frozen on dry ice, placed in Eppendorf tubes, and stored at −80°C until further processing. Analysis included grape skin tissues sampled during both seasons and pulp tissues sampled during the 2015 season.

### Analysis of skin phenylpropanoids

Grape skin samples were analyzed using Ultra Performance Liquid Chromatography coupled to a Quadrupole Time-of-Flight Mass-Spectrometer (UPLC QTOF-MS, Waters, MA, USA), following an extraction protocol for metabolite profiling as described in Weckwerth et al. (2004). Skin tissues were lyophilized and ground under liquid nitrogen using a RETSCH-mill (Retsch, Haan, Germany) with pre-chilled holders and grinding beads. The powder was weighed (40mg), and metabolites were extracted in a 1-ml pre-chilled methanol:chloroform:water extraction solution (2.5:1:1 v/v). Internal standards, i.e., 0.2 mg/ml ribitol in water, 1 mg/ml ampicillin in water, 1 mg/ml corticosterone in methanol, were subsequently added. The mixture was then briefly vortexed, and 100 μl of methanol was added; the mixture was then placed on a horizontal shaker for 10 min at 1000 rpm. The samples were later sonicated for 10 min (Elmasonic S30, Elma, Singen, Germany) and centrifuged for 10 min (20,817 × g, microcentrifuge 5417R, Eppendorf, Hamburg, Germany). The supernatant was decanted into new tubes, mixed with 300 μl of chloroform and 300 μl of MiliQ water (Millipore, MA, USA), vortexed for 10 s and then centrifuged at 20,817 × g for 5 min. Next, the water/methanol phase was separated, filtered (0.22 μm Millipore, MA, USA) and transferred to UPLC vials for analysis.

### LC/MS conditions

UPLC-QTOF-MS conditions were exactly as described previously by Hochberg et al. (2013).

### LC/MS annotation

MassLynxTM software (Waters) version 4.1 was used for system control and data acquisition. Metabolite annotation was validated using the standard libraries described in Arapitsas et al. (2012), based on retention time order, given in Degu et al. (2014). Metabolites were also annotated based on fragmentation patterns searched against the Chemspider metabolite database (http://www.chemspider.com/), the consistency of their retention times with those of identified metabolites, and comparison with the data in the current scientific literature.

### Analysis of pulp primary metabolites

Pulp samples were manually crushed with a mortar and pestle while kept frozen with liquid nitrogen. Next, 100 mg of crushed, frozen powder was weighed and extracted by adding a methanol:chloroform:water solution (2.5:1:1), similar to the aforementioned phenylpropanoid extraction. Then, 70 μl of the extract were dried using a Concentrator Plus (Eppendorf, Hamburg, Germany) and derivatized exactly as described in Hochberg et al. (2015) with sorbitol as the internal standard. Glucose, fructose, tartaric and malic acids were quantified using a calibration curve of standards (Sigma-Aldrich, MO, USA) with 10 concentration points from 50 to 900 ng for glucose and fructose and 2.5 to 45 ng for malic and tartaric acids. A split ratio of 50:1 was used to correctly determine the levels of glucose, fructose, malic and tartaric acids, due to their relatively high abundance in the pulp. The GC-MS conditions were exactly as described previously by Hochberg et al. (2013).

The Xcalibur data system V2.0.7 was used for system control and data acquisition. Annotation was based on spectral searching supported by the National Institute of Standards and Technology (NIST, Gaithersburg, MA, USA) against RI libraries from the Max-Plank Institute for Plant Physiology (Golm, Germany).

### Statistical analysis

Statistical analysis was performed using R v3.3.1 in RStudio. A profile analysis, from the “profileR” package, was used in order to compare the spatial profiles of clusters from exposed and shaded treatments and both canopy sides. This analysis tested the multivariate spatial data with two separate null hypotheses: a) the multivariate profile between groups is parallel; and b) the multivariate levels between groups are equal. This was done separately for each of the 70 annotated metabolites. In addition, within-cluster differences were tested for significance using the aov() function for ANOVA and the *post-hoc* Tukey test using the “agricolae” package. The same method was used for comparing the within-cluster coefficient of variance for each metabolite between different shading treatments, to highlight significant differences in cluster homogeneity caused by shading. Differences in malate/tartaric acid levels were tested for significance by using the built-in *t*-test() function.

The Pearson correlation of the “corrplot” package was used in order to construct separate metabolite correlation matrices for fully exposed and 60% shaded clusters, based on the entire set of samples from each treatment.

A regression analysis of each metabolite with daily incoming SI levels was done using the entire set of samples obtained from all treatments and cluster orientation, for which irradiance data was available. Linear and logarithmic regressions were assessed by the built-in lm() function, using non-transformed and log-transformed irradiance data, respectively. The *R*^2^-values of metabolites found to have significant regressions were then calculated by using the mean of four biological replicates, in order to minimize the effect of within-group variability on this coefficient.

Heatmap figures were generated using TMeV v4.9, using the mean of four biological replicates. Hierarchical clustering of the heatmaps was based on the Pearson correlation.

### Network analysis

Correlation networks were constructed based on the data obtained from Pearson correlation analyses performed in R (detailed above), separately for exposed and 60% shaded treatments, and for metabolites detected in pulp and skin tissues (i.e., primary and secondary metabolites). Visualization and computation of network properties were performed using the “MetScape” application and the NetworkAnalyzer tool, respectively, available in Cytoscape V3.4.0. Correlations were incorporated into the network if the *r*-value was *r* > 0.5 or *r* < (−0.5).

## Results

### The spatial distribution of micrometeorological conditions across the cluster differed in response to shading and position

Cluster-zone shading successfully filtered the incoming SI as shown in Table 1. Clusters shaded with 30% and 60% shading nets received 64% and 34%, on average, of the incoming SI measured for fully exposed clusters, respectively. This did not lead to measured differences in the cluster-zone air temperature and relative humidity between treatments (data not shown). Comparing the different orientations in the east-facing cluster, the east-south orientation (orientation 1) received the largest amount of daily SI, followed by east-north (orientation 2) and finally west-south (orientation 3). The higher SI recorded for east-north compared to west-south may be attributed to the sun's zenith relative to the row orientation at the time of direct sunlight exposure on the corresponding sensors.

**Table 1:** **Sums of daily irradiance by cluster orientation in the three shading treatments**.

**Orientation**	**Sum of daily irradiance (MJ/m^2^/day)**			**Relative sum of daily irradiance**		
	**Exposed**	**30% shaded**	**60% shaded**	**Exposed**	**30% shaded**	**60% shaded**
1	14.0	9.4	4.9	100%	100 (67.2)%	100 (35.2)%
2	10.1	6.7	3.4	72.4%	70.8 (65.7)%	68.4 (33.3)%
3	7.2	4.6	2.6	51.5%	48.5 (63.4)%	53.1(36.3)%
4	–	–	–	–	–	–
5	–	–	–	–	–	–
6	5.7	3.7^*^	1.9^*^	40.9%	38.9 (64)%	39.5 (34)%
7	11.2	7.2^*^	3.8^*^	80.2%	76.3 (64)%	77.4 (34)%
8	11.5	7.4^*^	3.9^*^	82.2%	78.2 (64)%	79.3 (34)%
Station	26.8					

Sums of daily irradiance, measured within the cluster-zone to represent the different cluster orientations.Values marked by

* *are estimated based on the mean filtering efficiency of each shading net. Relative sums of daily irradiance values represent the percentage compared to the daily irradiance in orientation 1 of each treatment separately. Values enclosed in parentheses represent the percentage compared to the daily irradiance of the exposed treatment for each orientation separately. Data were not collected for orientations 4 and 5*.

Interestingly, a comparison between the exposed and the 30% shaded clusters, and the 30% shaded and the 60% shaded clusters revealed that incoming SI differences within a cluster exceeded the differences between treatments. Finally, while the percentage of SI intercepted by the different cluster orientations, compared to the reference of each treatment, remained unaffected, lowering the overall incoming SI levels to the cluster, by shading, effectively minimized the within-cluster heterogeneity in SI. Shading reduced the incoming energy differences between orientations 1 and 3 by 3-fold, from 6.8 to 2.3 MJ/m^2^/day for exposed and 60% shaded clusters, respectively.

**Figure 2:**
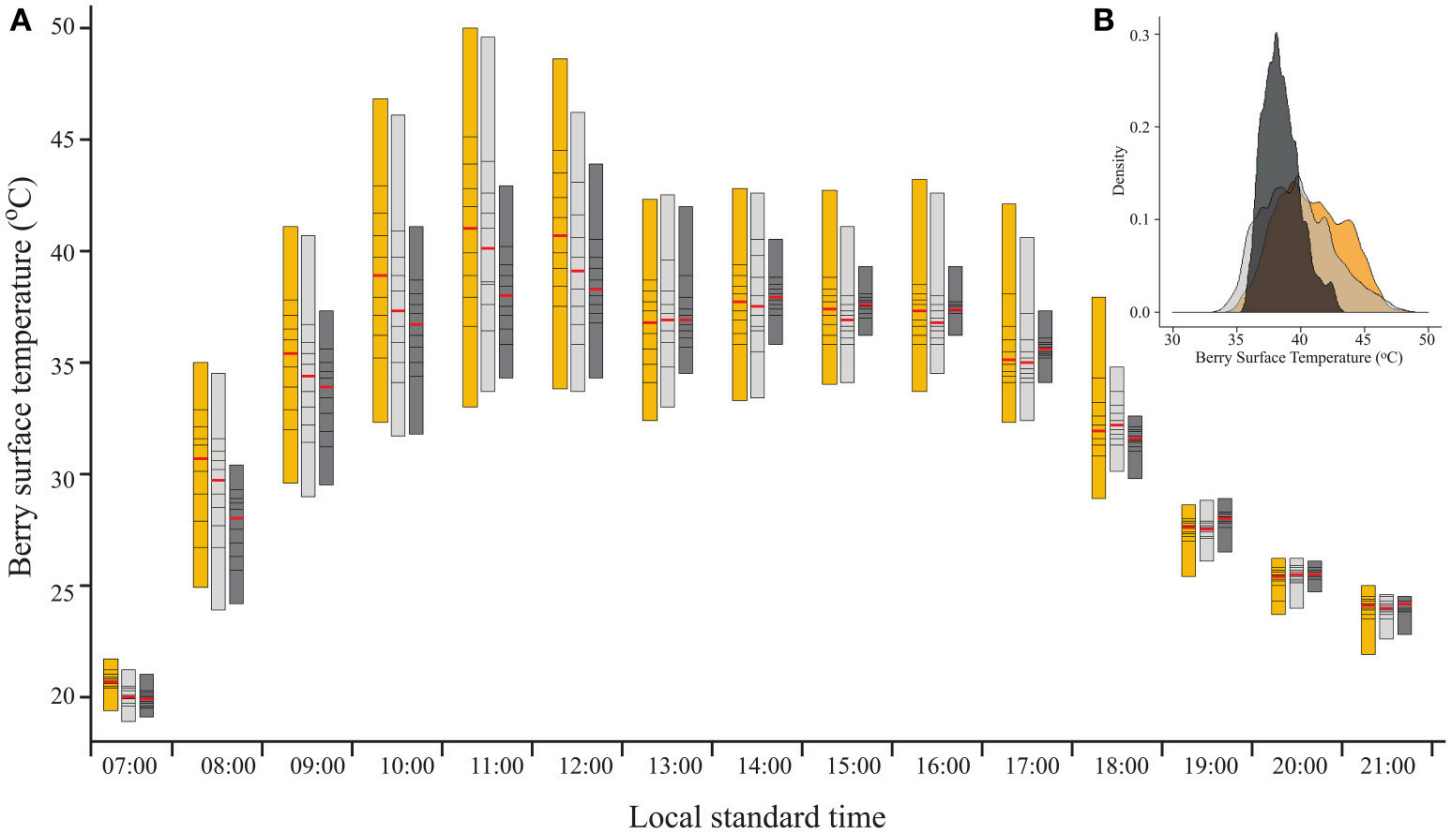
**(A)** Percentile bars showing the diurnal trend of berry surface temperature, measured on August 19, 2015 on clusters located on the east side of the canopy, subjected to three sunlight exposure levels: fully exposed clusters (exposed), clusters shaded with 30% shading nets (30% shaded) and clusters shaded with 60% shading nets (60% shaded). Red lines highlight the median values. **(B)** Density plot of the data shown for 11:00.

As expected, filtering the incoming SI caused a reduction in BST (Figure 2A). As shown by the percentiles, during direct sun exposure hours (e.g., morning for east-facing clusters), shading reduced the maximum BST, while having no effect on the minimum values, which represent the temperature of the shaded cluster faces. Notably, during the afternoon, when the east-facing clusters were exposed only to diffused radiation, the minimum BST of the 60% shaded clusters increased, while it remained stable for the 30% shaded and fully exposed clusters. Shading clusters with 60% shading nets decreased their maximum temperature by up to 7.1°C and daytime (6:00–20:00) mean BST by 0.7°C (Table 2). Examining the distribution of BST within a cluster, presented in histograms (Figure 2B), revealed that shaded clusters were more homogeneous than exposed ones. While maximum differences in BST within an exposed cluster reached 17°C (33 to 50°C at 11:00), they were 9.6°C in 60% shaded clusters (34.3 to 43.9°C at 12:00).

**Table 2:** **Berry surface temperature by cluster orientation in the three shading treatments**.

**Orientation**	**Mean of daily BST (°C)**			**Maximum daily BST (°C)**		
	**Exposed**	**30% shaded**	**60% shaded**	**Exposed**	**30% shaded**	**60% shaded**
1	28.0	27.6	27.8	50.0	49.6	43.9
2	27.5	26.9	27.6	46.8	45.9	42.2
3	27.9	27.5	27.7	48.2	48.8	43.0
4	–	–	–	–	–	–
5	–	–	–	–	–	–
6	26.2	25.1	26.7	45.5	41.3	41.8
7	27.1	25.9	27.0	48.3	43.9	44.6
8	26.6	25.7	26.8	48.7	43.9	42.9
Station	25.8			33.4		

*Berry surface temperature (BST) calculations based on hourly spatial measurements of representative clusters acquired on August 19, 2015. Data were not collected for orientations 4 and 5*.

### Cluster spatial profile of metabolite level was affected by SI intensity and cluster position

**Figure 3:**
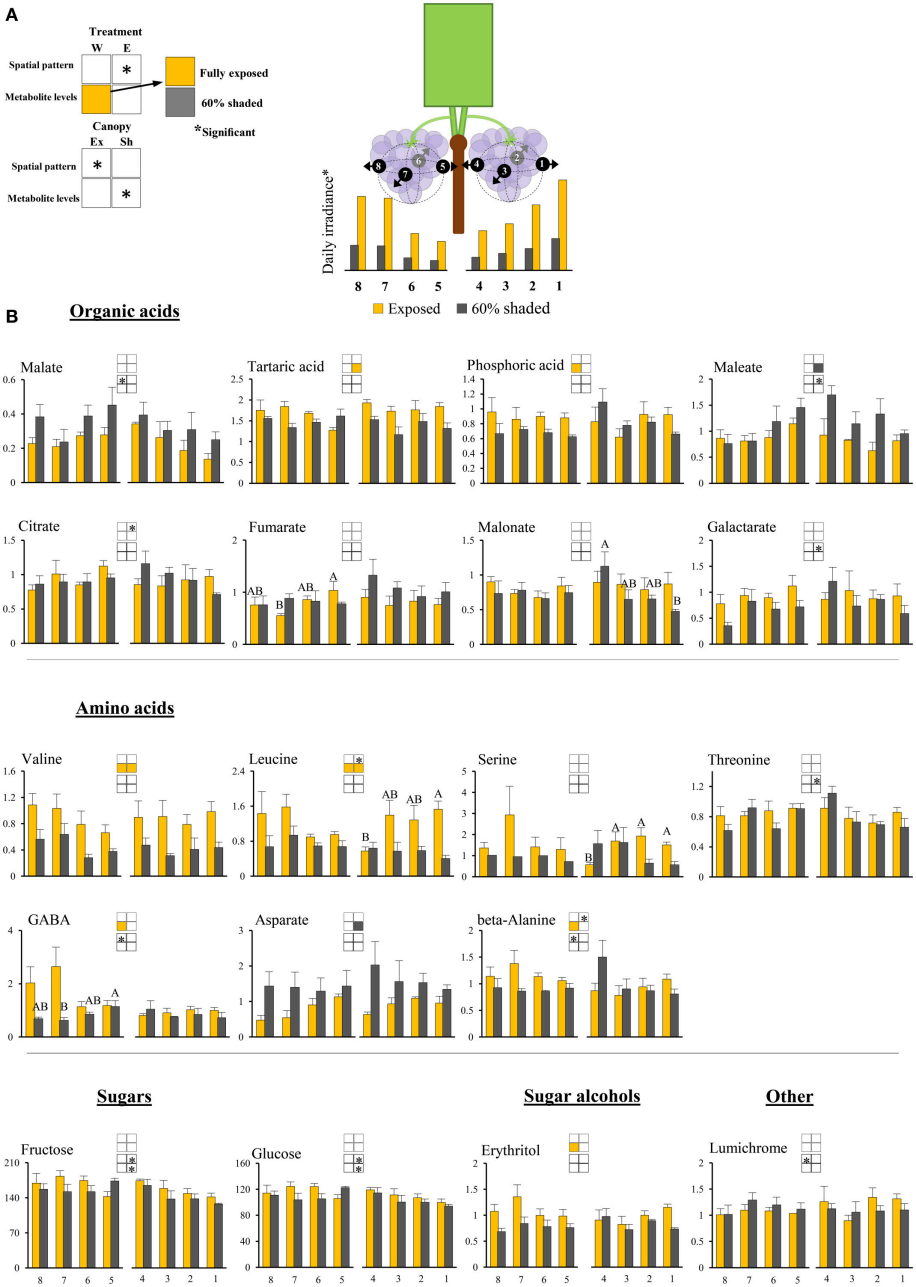
**Cluster spatial level results for primary metabolites detected by GC-MS in berry pulp tissues sampled from different cluster orientations and shading treatments. (A)** Illustrations of the daily irradiance levels in each cluster orientation including estimations based on diffusive light measurements and the measured mean filtering capacity of the shading nets. **(B)** Organic acids, amino acids, sugars, sugar alcohols, and other compounds found to be significantly affected by SI levels, cluster position and/or cluster orientation. Levels represent relative abundance based on ion count. Numbers on the X axis represent cluster orientations. Yellow bars represent fully exposed clusters, and dark gray bars represent 60% shaded clusters. Error bars are standard error (*n* = 4). Bars of the same cluster location and treatment, marked by different letters, represent significantly different values (α < 0.05). Information given in the boxes details the significant effects of treatment (upper box) for clusters located on the east (E) and the west (W) side of the canopy, as well as canopy side (lower box), on the spatial pattern and profile levels of metabolite accumulation in fully exposed (Ex) and 60% shaded (Sh) clusters. Asterisks in upper box indicate a significant effect of treatment on the spatial pattern of compound accumulation. Yellow and gray boxes indicate a significantly higher overall compound level in the exposed and 60% shaded treatments, respectively. Asterisks in lower boxes indicate a significant effect of canopy side on the spatial pattern and overall compound levels.

**Figure 4:**
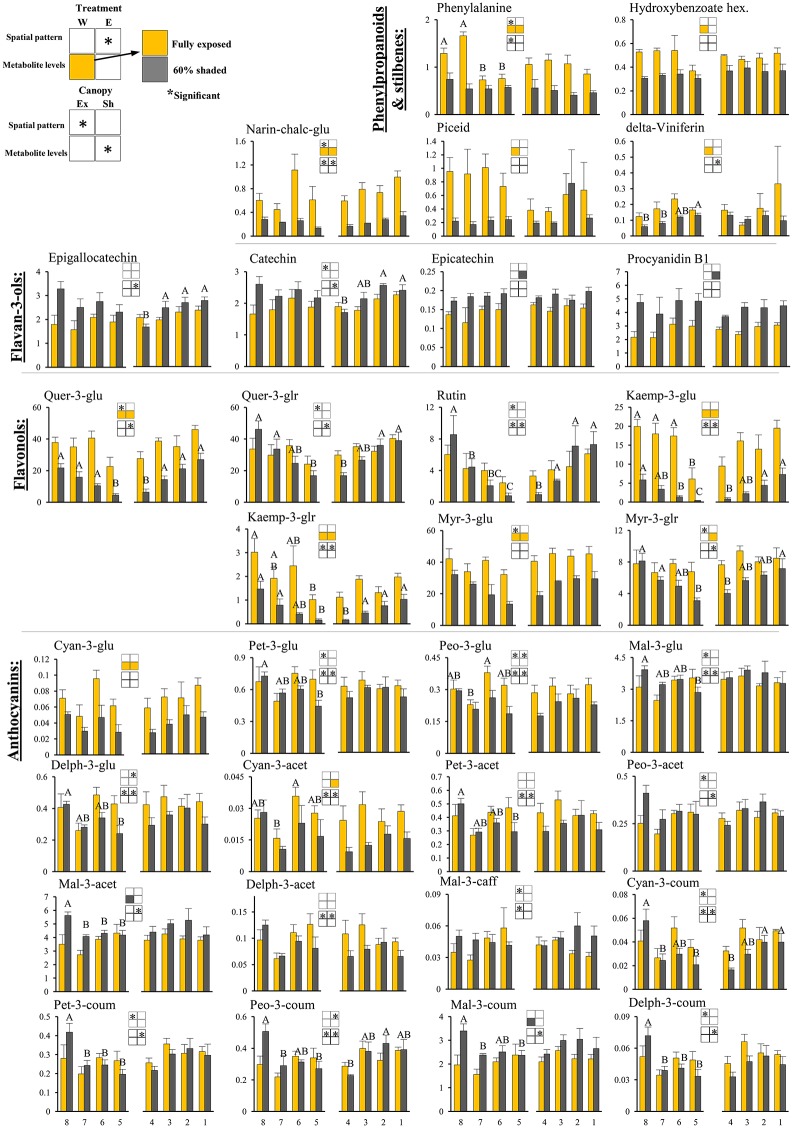
**Cluster spatial level results for phenylpropanoid metabolites detected by UPLC-QTOF-MS in berry skin tissues sampled from different cluster orientations and shading treatments**. Included are pivotal phenylpropanoids and stilbenes, flavan-3-ols, flavonols, and anthocyanins found to be significantly affected by SI levels, cluster position and/or cluster orientation. Levels represent relative abundance based on ion count. Numbers on the X axis represent cluster orientations. Yellow bars represent fully exposed clusters, and dark gray bars represent 60% shaded clusters. Error bars are standard error (*n* = 4). Bars of the same cluster location and treatment, marked by different letters, represent significantly different values (α < 0.05). Information given in the boxes details the significant effects of treatment (upper box) for clusters located on the east (E) and the west (W) side of the canopy, as well as canopy side (lower box), on the spatial pattern and profile levels of metabolite accumulation in fully exposed (Ex) and 60% shaded (Sh) clusters. Asterisks in upper box indicate a significant effect of treatment on the spatial pattern of compound accumulation. Yellow and gray boxes indicate a significantly higher overall compound level in the exposed and 60% shaded treatments, respectively. Asterisks in lower boxes indicate a significant effect of canopy side on the spatial pattern and overall compound levels. Illustrations of the daily irradiance levels in the corresponding cluster orientation are given in Figure 3A.

The spatial profile analysis, i.e., the levels and pattern of metabolite accumulation in response to the berry position/orientation, was conducted separately for the primary (Figure 3) and secondary metabolites (Figure 4), and summarized in Supplementary Table 1.

#### The impact of cluster shading on the spatial pattern and overall levels of fruit metabolites

##### Pulp primary metabolites

Shading affected the spatial pattern (i.e., the pattern of accumulation in the different cluster orientations) of leucine, beta-alanine, and citrate in east-located clusters, increasing from internal to external orientations in exposed clusters, while shaded clusters had the opposite trend. Shading increased the levels of maleate and aspartate by 1.6- and 1.8-folds, respectively, and decreased the levels of tartaric acid, valine, and leucine by 1.3-, 2.2-, and 2.2-folds, respectively, in east-located clusters. In west-located clusters, shading decreased the levels of phosphoric acid, erythritol, beta-alanine, valine, leucine, and GABA by 1.3-, 1.4-, 1.3-, 1.9-, 1.6-, and 2.1-folds, respectively.

##### Skin phenylpropanoids

Shading significantly affected the levels of 11 and 12 phenylpropanoids in the east- and west-located clusters, respectively, in 2015. Shading increased the levels of procyanidin B1 and epicatechin by 1.5- and 1.2-folds, respectively, and decreased the levels of the anthocyanins cyan-3-glu and cyan-3-acet (1.8- and 2-folds, respectively), phenylalanine and narin-chalc-glu (2.1- and 3-folds), and the flavonols myr-3-glu, myr-3-glr, quer-3-glu, kaemp-3-glu, and kaemp-3-glr by 1.7-, 1.4-, 2.1-, 4-, and 2.5-folds, respectively, in east-located clusters. In west-located clusters, shading significantly increased the levels of both mal-3-acet and mal-3-coum by 1.3-fold, and decreased the levels of cyan-3-glu, phenylalanine, narin-chalc-glu, and hydroxybenzoate hex (1.8-, 1.8-, 3-, and 1.5-folds), the stilbenes delta-viniferin and piceid (1.8- and 4.2-folds), and the flavonols myr-3-glu, quer-3-glu, kaemp-3-glu and kaemp-3-glr by 1.6-, 2.6-, 5.6-, and 2.8-folds, respectively.

#### The impact of cluster canopy side on the spatial pattern and overall levels of fruit metabolites

##### Pulp primary metabolites

The canopy side of the cluster significantly affected the spatial pattern of malate, galactarate, maleate, threonine, glucose, and fructose in shaded clusters, while no significant effect was found in exposed clusters (Figure 3B). All mentioned metabolites followed a trend in which external cluster orientations had lower values than the internal ones, resulting in an opposite east-north-south-west pattern between the two sides of the canopy, corresponding with SI. In addition, canopy side significantly affected the overall levels of glucose and fructose in shaded clusters, and of lumichrome, beta-alanine, and GABA in exposed clusters. With the exception of lumichrome, all were found to be higher in clusters from the west side of the canopy than in those from the east.

##### Skin phenylpropanoids

Canopy side affected the spatial profile of 15 and 24 phenylpropanoid metabolites in exposed and shaded clusters, respectively, in the 2015 season (Figure 4). These included all the anthocyanins, with the exception of cyan-3-glu and vitisin A, the flavan-3-ols epigallocatechin and catechin, narin-chalc-glu and phenylalanine, and the entire set of flavonols, with the exception of myr-3-glu. In the 2014 season, canopy side significantly affected the spatial profile of only four and five phenylpropanoid metabolites, in exposed and shaded clusters, respectively; of these, mal-3-glu and peo-3-coum, in exposed clusters, and the flavonols rutin, quer-3-glr, quer-3-glu, and kaemp-3-glu, in shaded clusters, repeated in both years. Canopy side had no significant effect on the levels of phenylpropanoids in 2015, while in 2014, mal-3-glu was higher in the east-located clusters than in the west; the opposite was found for phenylalanine.

Overall, the proportion of metabolites showing significant responses to canopy side and shading were higher in the phenylpropanoids than in the primary metabolites, with sugars and hydroxy-cinnamates being the least affected chemical groups in pulp and skin tissues, respectively.

### Common and differential responses of metabolite groups to SI

**Figure 5:**
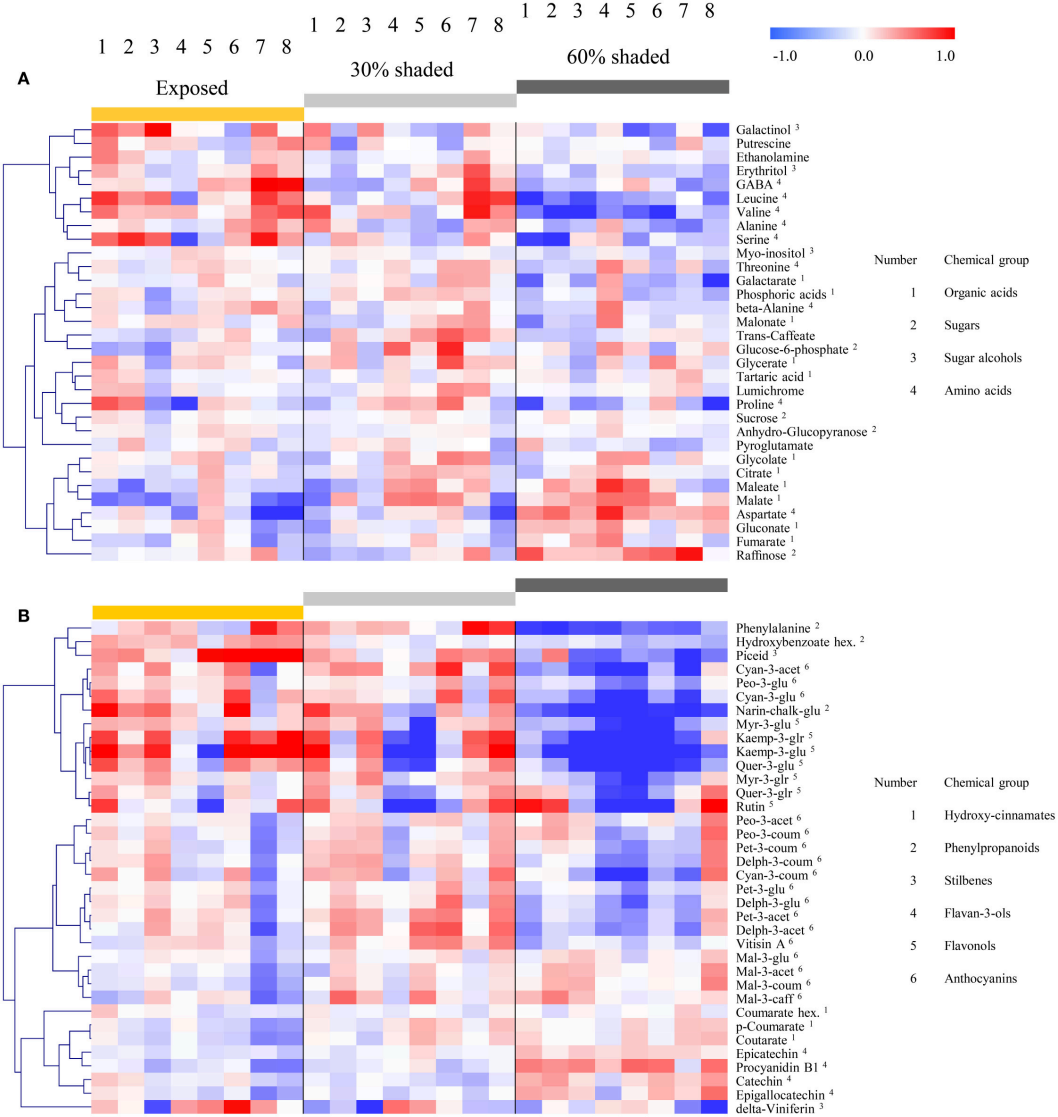
**Heatmap of grape pulp primary metabolites (A)** and skin phenylpropanoids **(B)** across eight orientations (see Figure 1) of clusters subjected to three sun exposure treatments: fully exposed clusters (exposed), clusters shaded with 30% shading nets (30% shaded) and clusters shaded with 60% shading nets (60% shaded). The heatmap was generated using the mean values of four biological replicates following normalization to the median of each metabolite based on all samples, and log2 transformation. The Pearson correlation was used for hierarchical clustering of the metabolites.

Hierarchical clustering of primary metabolites (Figure 5A) highlighted three major compound groups. The first group was composed mainly of nitrogenous compounds, including the amino acids leucine, valine, alanine, serine, and GABA, the biogenic amines putrescine and ethanolamine, and two sugar alcohols, galactinol, and erythritol. This group's compounds showed an increase from shaded to fully exposed clusters. In addition, in 30% shaded and fully exposed clusters, a generally higher abundance was found in external cluster orientations (i.e., facing the inter-row), especially for clusters located at the west side of the canopy, than in the internal orientations (i.e., facing the canopy), as seen for orientations 7 and 8 (external) compared to 4 and 5 (internal). The second group included metabolites associated with the TCA cycle, such as citrate, fumarate, malate, as well as the closely linked aspartate and maleate, in addition to glycolate, gluconate, and raffinose. These compounds showed a gradual decrease from the densely shaded to the fully exposed samples. In addition, their content was greater in the internal cluster orientations than in the external ones, irrespective of the treatment, as seen for orientations 4 and 5 compared to 1 and 8, respectively. The third group, comprising tartaric acid, the amino acids proline, beta-alanine and threonine, sucrose, glucose-6-phosphate, phosphoric acids, galactarate, malonate, trans-caffeate, pyroglutamate, anhydro-gluopyranose, myo-inositol, and lumichrome, had a less pronounced pattern of change between treatments and cluster orientations.

Clustering the phenylpropanoids using the 2015 season data (Figure 5B) grouped together compounds according to their chemical properties and biochemical pathways, such as those belonging to the flavonols, flavan-3-ols and hydroxy-cinnamates. In general, the flavonols showed a strong increase from the densely shaded to the exposed clusters, while the flavan-3-ols and cinnamates had the opposite trend. In contrast to the mentioned groups, the anthocyanins showed metabolite-specific trends. Cyanidin (glycosylated and acetylated) and peonidin (glycosylated) increased from the densely shaded to the exposed clusters and were grouped with the flavonols, as well as phenylalanine and the stilbene piceid, exhibiting an opposite trend to that of the malvidin metabolites. The rest of the anthocyanins were grouped together and exhibited an optimum in the 30% shaded cluster samples.

The 2014 season data (Supplementary Figure 4), based on fully exposed and 60% shaded clusters, yielded similar trends. However, in contrast to the 2015 results, in the 2014 season, the coumaroylated forms of cyaniding, delphinidin, and petunidin clustered with the flavonols, while the glycosylated cyanidin and peonidin, together with the majority of the anthocyanins, formed a separate group.

### Within-cluster/within-vine heterogeneity of metabolite abundance was affected by exposure to SI

To study how the within-cluster heterogeneity of metabolite content was affected by shading, metabolite abundance values in samples taken from different orientations of an individual cluster were normalized to that specific cluster median. A hierarchical clustering of the samples (i.e., based on cluster orientations) from the 2015 season (Supplementary Figure 5) was used as a qualitative assessment of cluster heterogeneity where the magnitude of change from the cluster median is represented by the intensity of red and blue colors. Spatial patterns common to all treatments were evident, as samples from the internal orientations (i.e., samples 4 and 5) were clustered together and apart from the external ones (i.e., samples 1 and 8). Among the primary metabolites (Supplementary Figure 5A), organic and amino acids were the most heterogeneous compounds in all treatments, in contrast to the sugars glucose, fructose, and sucrose that were relatively uniform across cluster orientations. Finally, a subtle gradual increase in cluster heterogeneity was visible from the more uniform 60% shaded clusters to the more heterogeneous exposed clusters for the nitrogenous compounds putrescine, pyroglutamate, and ethanolamine, and the compounds trans-caffeate, lumichrome, and erythritol. Among the different phenylpropanoid groups (Supplementary Figure 5B), the flavonols were the most heterogeneous; furthermore, their within-cluster variability clearly increased with increasing levels of shading.

To further verify this trend, the coefficient of variance of each metabolite was calculated per vine (i.e., within all samples originating in a single vine). Values were compared between treatments, and a significant treatment effect on the coefficient of variance was found for five out of the total 70 metabolites (Supplementary Figure 6). Among the primary metabolites, putrescine had a significantly lower coefficient of variance in the 60% shaded clusters than in the 30% shaded and fully exposed clusters, showing an improvement in uniformity with shading. In the phenylpropanoid group, the glucuronide and glycosylated forms of the flavonols quercetin and kaempferol had a significantly higher coefficient of variance in the 60% shaded clusters than in the 30% shaded and fully exposed clusters, showing an improvement in cluster uniformity with an increasing degree of sunlight exposure.

### Modulation of metabolite-specific abundance by sunlight

**Figure 6:**
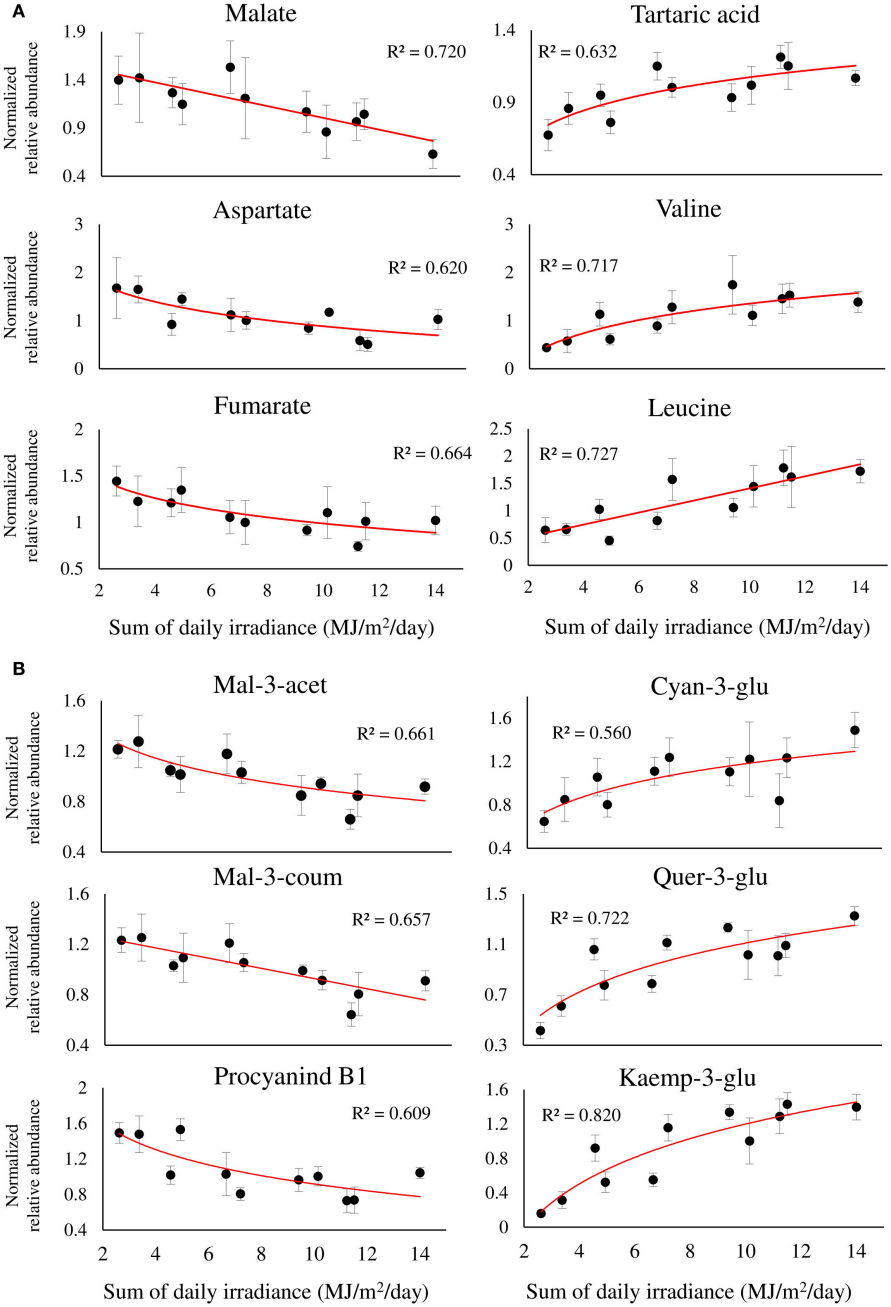
**Twelve selected metabolites of primary (A)**, and phenylpropanoid **(B)** metabolism, showing a significant linear or logarithmic regression with the sum of daily irradiance across the dataset, obtained from different orientations and locations of grape clusters subjected to three sun exposure treatments: fully exposed clusters (exposed), clusters shaded with 30% shading nets (30% shaded) and clusters shaded with 60% shading nets (60% shaded). *R*^2^ values are based on a regression of means of four biological replicates. Regression analysis was based on the entire dataset (α < 0.05, *n* = 4).

The metabolite profiles of berries exposed to a gradient of SI intensity resulted in a significant linear regression between 33, out of 70, metabolites and the sum of daily SI. Figure 6 shows the regression of 12 metabolites of interest that obtained *R*^2^ > 0.5, highlighting major metabolic shifts in the fruit in response to SI. Among the primary metabolites (Figure 6A), the two most abundant organic acids, malate and tartaric acid, were found to have contrasting responses to incoming SI levels, showing negative and positive regressions with incoming SI levels, respectively. This caused a significant increase in the ratio of tartaric acid/malate in fully exposed clusters compared to shaded ones (Supplementary Figure 7). As with malate, fumarate and aspartate showed similar negative responses. In contrast, a large number of nitrogenous compounds, including beta-alanine, the branch-chained amino acids, valine and leucine, stress-related proline and GABA, and the biogenic amines, putrescine and ethanolamine, had positive regressions with SI levels (Figure 6A and Supplementary Table 2).

Among the phenylpropanoids (Figure 6B and Supplementary Table 2), the flavan-3-ols catechin, epicatechin, epigallocatechin, and procyanidin B1 had negative regressions with SI levels. A similar negative trend was found for malvidin anthocyanins, and acetylated and coumaroylated peonidin, while cyanidin-glucoside showed a contrasting response, verifying the preceding results shown in Figure 5B. As expected, the flavonols showed positive regressions with incoming SI. In addition, the logarithmic trend that best fitted the response of the glycosylated kaempferol and quercetin was in accordance with the higher variability of these metabolites found by within-cluster hierarchical clustering (Supplementary Figure 5), and calculated as an increase in the coefficient of variance, observed under low-light conditions (i.e., 60% shaded clusters) in Supplementary Figure 6.

### Metabolic coordination in response to SI differed between exposed and shaded clusters

**Figure 7:**
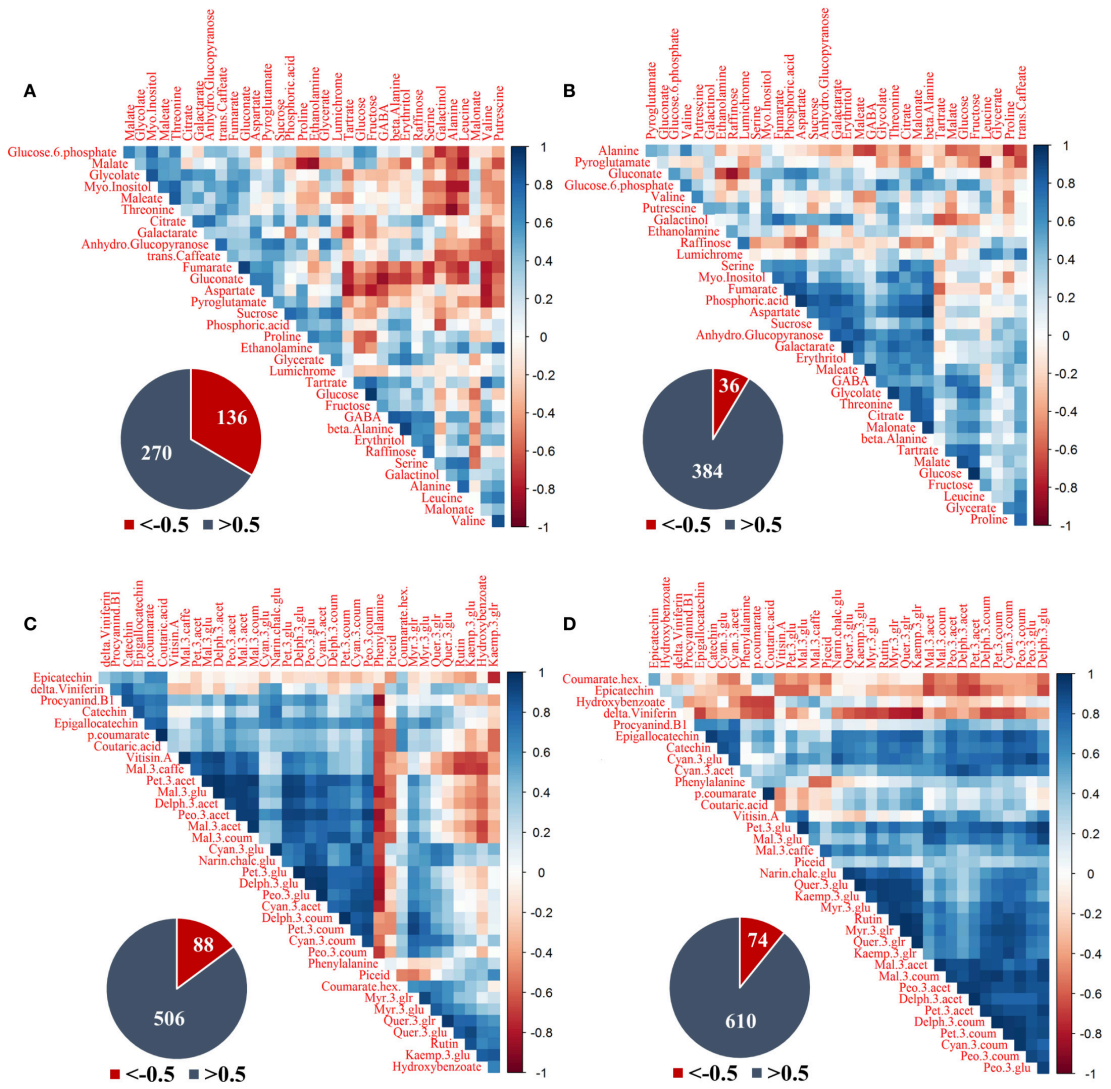
**Correlation analysis of grape pulp primary metabolites and skin phenylpropanoids of clusters subjected to two sun exposure treatments: fully exposed clusters (exposed) and clusters shaded with 60% shading nets (60% shaded). (A)** Primary metabolites of exposed, **(B)** primary metabolites of 60% shaded, **(C)** phenylpropanoids of exposed, and **(D)** phenylpropanoids of 60% shaded. The analysis was generated using the Pearson correlation on the mean values of four biological replicates. The corresponding pie charts show the number of negative correlations (*R* < −0.5, in red) compared to positive correlations (*R* > 0.5, in blue) within the correlation matrices.

Correlation matrices were separately constructed for primary and phenylpropanoid metabolites based on samples obtained during the 2015 season, from all cluster orientations and canopy sides of the same treatment. This allowed a comparison of the metabolite coordination of exposed vs. 60% shaded clusters in response to changes in SI (Figures 7A–D). For both metabolite groups, exposed clusters had a higher number of negative correlations between metabolites and a lower number of positive correlations compared to the 60% shaded clusters. The same trend was found in the phenylpropanoid data obtained in 2014.

Primary metabolites (Figures 7A,B) had 136 negative correlations with *r* < −0.5 in exposed clusters compared to 36 in the 60% shaded clusters, and 270 positive correlations with *r* > 0.5 compared to 384, respectively. In the exposed clusters, the amino acids serine, alanine, leucine and valine, the biogenic amine putrescine, and galactinol were found to negatively correlate with glucose-6-phophate, glycolate, myo-inositol, glucopyranose, trans-caffeate, and gluconate, as well as with malate, fumarate, maleate, aspartate, and threonine. Fumarate, gluconate and aspartate were also negatively correlated with tartaric acid, glucose, fructose, GABA, beta-alanine, erythritol, and raffinose. Tartaric acid and galactarate were negatively correlated in addition to a strong negative correlation of proline and ethanolamine with malate. GABA, beta-alanine and erythritol were strongly positively correlated, as well as glucose and fructose, fumarate and gluconate and the group of glucose-6-phosphate, malate, glycolate, maleate, myo-inositol, and threonine. In the 60% shaded clusters, alanine was negatively correlated with the associated citrate, malate and maleate, the amino acids GABA and proline and trans-caffeate. Pyroglutamate had a strong negative correlation with leucine, while gluconate was negatively correlated with raffinose. In contrast, a large group of metabolites, comprising serine, threonine, aspartate, GABA, myo-inositol, fumarate, phosphoric acid, maleate, glycolate, citrate, malonate, galactarate, sucrose, glucopyranose, and erythritol, were positively correlated, in addition to a strong positive correlation between tartaric acid, malate and glucose.

The phenylpropanoids (Figures 7C,D) shared 88 negative correlations with *r* < −0.5 in exposed clusters compared to 74 in the 60% shaded clusters and 506 positive correlations with *r* > 0.5 compared to 610, respectively. In the exposed clusters, phenylalanine was found to negatively correlate with a large number of metabolites, including all the annotated flavan-3-ols and anthocyanins, and to positively correlate with the flavonols of kaempferol, as well as quercetin conjugates (excluding quer-3-glr). The latter flavonols, in addition to hydroxy-benzoate, were negatively correlated with a large number of anthocyanins, including all malvidin metabolites and the acetylated forms of peonidin, petunidin and delphinidin, but not with their glycosylated and coumaroylated forms, nor with any of the cyanidin metabolites. In contrast, in the 60% shaded clusters, phenylalanine showed only few, weak negative correlations, and correlations between flavonols and anthocyanins were strongly positive. Instead, epicatechin and coumarate hex showed differing degrees of negative correlations with the entire set of annotated anthocyanins. Finally, correlations between the stilbene delta-viniferin and flavonols, which were slightly positive in the exposed clusters, were strongly negative in the shaded clusters.

Four networks were created based on the correlation matrices of the four datasets (Supplementary Figures 8A–D), two treatments (exposed and 60% shaded clusters), and two metabolic groups (primary and phenylpropanoid metabolites), across the different cluster locations and orientations. At *r* > 0.5 and *r* < −0.5, shading slightly increased the number of edges in the primary metabolite network from 157 to 169, the network density from 0.28 to 0.301, the clustering coefficient from 0.486 to 0.537 and the average node degree from 9.24 to 9.94. In the phenylpropanoid networks, shading increased the number of edges from 303 to 340, the network density from 0.481 to 0.54, the clustering coefficient from 0.71 to 0.77 and the average node degree from 16.83 to 18.89. Shading caused an at least 2-fold increase in the nodal degree of the primary metabolites glycolate, galactarate, phosphoric acid, maleate, malonate, beta-alanine, and fructose, and an at least 2-fold decrease in the nodal degree of the nitrogenous compounds valine, serine, leucine, proline, putrescine, ethanolamine and pyroglutamate, as well as glycerate and tartaric acid. In the phenylpropanoids, shading caused an at least 2-fold increase in the nodal degree of the flavonols quer-3-glu, rutin, kaemp-3-glu, and kaemp-3-glr, the flavan-3-ols catechin and Epicatechin, and the stilbene delta-viniferin, and an at least 2-fold decrease in the nodal degree of phenylalanine, the hydroxy-cinnamates p-coumarate and coutarate, hydroxy-benzoate hex, procyanidin B1, vitisin A, and the stilbene piceid.

## Discussion

The accumulation levels of the major primary and secondary metabolites that determine a grape berry's sensorial profile vary as a function of the micrometeorological conditions in its immediate vicinity (Jackson and Lombard, 1993; Downey et al., 2006). Nevertheless, few studies have investigated how these conditions vary within a vine and across a single grape cluster, and how their variation affects the spatial pattern of the fruit metabolic profile in the vineyard and crop uniformity at harvest. This study provided a detailed spatial characterization of metabolite abundance in clusters located on both canopy sides and subjected to different degrees of sun exposure. The coupling of this sampling layout with high resolution measurements of both SI and BST was used to assess the role of sun exposure as a determining factor in the spatial pattern and variability in grape clusters' chemical composition, and to expand current knowledge regarding compound-specific responses and plasticity to SI.

SI filtering was proven to be an efficient tool to minimize the within-cluster variability of both incoming irradiance and BST. Artificial shading resulted in decreased BST of sunlit berries and an increased BST of shaded berries. This phenomenon could not be explained by diffusive light or air temperature, since the diffusive light intensity was lower in the shaded treatment and air temperature within the cluster-zone showed no differences (data not shown). For both SI and BST, differences between the orientations of the same cluster exceeded those between treatments, stressing the extent of within-cluster variability and the range of conditions that can be investigated within a single grape cluster. Finally, due to the strong correlation between SI and BST, as previously suggested (Smart and Sinclair, 1976) and found here, and since SI plays an important role in both the direct triggering of fruit-located photoreceptors and in fruit thermal balance, SI was selected as the explanatory variable in this work.

Elevated fruit temperatures have previously been suggested to increase the metabolic flux toward the TCA cycle and to modify its regulation, utilizing malate to enhance its anaplerotic capacity in grapevine berries (Sweetman et al., 2014; Rienth et al., 2016). The former work related the flux increment to the enhanced biosynthesis of pyruvate (valine, leucine, serine and glycine), oxaloacetate (aspartate, threonine and isoleucine) and 2-oxoglutarate-driven compounds (GABA, proline and putrescine). Indeed, increased TCA flux and the accumulation of all or part of the mentioned metabolites have been described in response to a wide array of abiotic stresses in a number of plant species, as summarized by Krasensky and Jonak (2012) and Obata and Fernie (2012). Here, SI conditions (i.e., cluster location and sun exposure levels) triggered responses in malate and oxaloacetate-associated compounds (malate, fumarate and aspartate) that contrasted with those in pyruvate (valine, leucine and serine) and 2-oxoglutarate-derived (proline, GABA and putrescine) nitrogenous compounds. Increased SI levels involve a concomitant increase in tissue temperature, and their combination is expected to exert oxidative stress (Foyer et al., 1994). Indeed, redox homeostasis was recently suggested to be the link between the metabolic modulation of grapevine berries, found in different leaf-removal studies (Young et al., 2016). This may explain the increase in the levels of proline, GABA and the polyamine putrescine, which share a role in mitigating oxidative stress (Krasensky and Jonak, 2012). Furthermore, the decrease in TCA cycle metabolites and the increase in specific amino acids may also have resulted from an arrest in glycolytic activity coupled with protein degradation, generating amino acids in a non-biosynthetic manner (Araujo et al., 2011; Lehmann et al., 2012; Obata and Fernie, 2012). Unfortunately, current knowledge is lacking regarding the direct effect of SI on fruit primary metabolism. Hence, such metabolic shifts are currently attributed to the secondary effects of temperature, UV-B irradiance and combined stress responses. Future research on the photo-receptor-mediated modulation of primary metabolism can greatly contribute to our understanding.

When considering the results of the network analysis, a higher coordination of the metabolic processes in the central metabolism was observed in this study in shaded grapes. Compared to fully exposed grapes, glycolysis, the TCA cycle, and amino acid metabolism shared a greater number of correlations, and the relations were mainly positive. The fact that the acclimation of the exposed and shaded clusters to perturbations in SI levels resulted in opposite types of correlations between nitrogen and carbon metabolites points to a differential coordination, possibly resulting from a comparison between non-stress (shaded clusters) and stress-related (fully exposed) responses. As described above, and as shown previously in potted grapevines and grape cell cultures subjected to short-term heat treatment (Sweetman et al., 2014; Ayenew et al., 2015), and berries of vines exposed to water deficit (Hochberg et al., 2013), a combined stress response could lead to the specific accumulation of nitrogen metabolites. In contrast, a positive coordination was maintained between glycolysis sugars and TCA cycle intermediates in both conditions, emphasizing the tight co-regulation existing between these two metabolic pathways.

The induction of the flavonoids biosynthesis by fruit-located photo-receptors was shown in a number of species, including grapevine, as reviewed in Zoratti et al. (2014). Summarizing a large number of studies, it can be concluded that while the R2R3 MYB transcription factors family includes both positive and negative regulators of the flavonoid biosynthesis, light seemed to strictly induce the expression of positive ones. However, a more complex interaction is evident, considering the consequent increase in fruit temperature. As found by a number of studies (Spayd et al., 2002; Mori et al., 2007; Tarara et al., 2008; Azuma et al., 2012), above a certain threshold, temperature causes a reduction in the anthocyanin levels, possibly through degradation, whether enzymatic or non-enzymatic (Vaknin et al., 2005; Mori et al., 2007; Chassy et al., 2015). This results in an expected antagonistic effect of elevated BST and strong SI conditions, as found in the exposed berries. Under these assumptions, the response of anthocyanins to increasing SI levels is expected to reflect the metabolite turnover between biosynthesis and degradation. The negative linear effect of SI on the levels of acylated peonidin and malvidin and mal-3-glu, measured in this study, might not be solely attributed to the consequent increase in temperature, as the proportion of acylated anthocyanins was found to increase with rising temperatures (Downey et al., 2004; Tarara et al., 2008), and malvidin metabolites were found to be exceptionally stable to high temperatures in Mori et al. (2007). Instead, considering the contrasting linear increase in the levels of cyanidin-glucoside, it is possible that the combined heat and light modulation of the biosynthesis-related genes' expression patterns, as found in Azuma et al. (2012), was involved in the preferential biosynthesis of upstream metabolites as was observed for cyanidin-glucoside. This may explain the overall positive net turnover of cyanidin-glucoside, while downstream metabolites, such as acylated peonidin and malvidin, showed a clear negative result.

The induction of flavonol accumulation by increasing SI levels, measured here, is in accordance with a large number of studies regarding light-induced flavonol biosynthesis in grape berries (Czemmel et al., 2009; Matus et al., 2009; Carbonell-Bejerano et al., 2014; Liu et al., 2015) and overall accumulation (Haselgrove et al., 2000; Downey et al., 2004; Cortell and Kennedy, 2006; Pereira et al., 2006; Matus et al., 2009; Azuma et al., 2012), yet the regression between SI and flavonol-glucoside accumulation in grape skins has been found so far to be purely linear (Haselgrove et al., 2000). The extensive gradient and relatively high SI intensity present in our study may be the cause for this discrepancy, yet whether this trend is due to a stagnation in biosynthesis or to an increase in temperature-driven degradation remains unclear.

The accumulation of monomeric and condensed Flavan-3-ols in grape skin peaks around veraison, followed by a decrease during the final stages of ripening (Downey et al., 2004; Fujita et al., 2007; Cohen et al., 2012). Increased SI exposure was found to increase flavan-3-ols levels at veraison, yet, since sunlit berries showed a faster decline during ripening, differences were no longer evident at harvest (Downey et al., 2004; Fujita et al., 2007). In this study, conducted between veraison and harvest, the linear negative effect of SI on the levels of flavan-3-ols metabolites was in accordance with the cited literature. A possible explanation could be a delay in the typical post-veraison decrease in the expression of the biosynthesis-related genes LAR and ANR under shading, as found in Fujita et al. (2007).

The stilbenes, which play a role in antifungal activity (Langcake, 1981; Pont and Pezet, 1990; Pezet et al., 2004) and offer potential health promoting effects (Bradamante et al., 2004; Baur et al., 2006; Gresele et al., 2011), accumulate in grapevine tissues in response to biotic and abiotic stresses, such as mold development, wounding, and UV-C irradiation treatments (Fritzemeier and Kindl, 1981; Bais et al., 2000; Vannozzi et al., 2012). Attempts to understand the impact of environmental factors on the accumulation of stilbenes in grape skin tissues have yielded confounding results, possibly owing to the importance of genetic, developmental, and pedological factors (Versari et al., 2001; Bavaresco et al., 2007, 2008, 2012; Berli et al., 2008; Carbonell-Bejerano et al., 2014; Degu et al., 2016). In this study, no significant correlation was found between SI and the levels of the stilbenes piceid and delta-viniferin. However, their levels were significantly higher in the exposed, compared to the shaded, clusters that were positioned on the west side of the canopy but not the east, an opposite trend to that of the major anthocyanins Mal-3-acet and Mal-3-coum. Considering the UV-B induction of stilbene synthase (STS) expression and stilbene accumulation (Versari et al., 2001; Carbonell-Bejerano et al., 2014), and the fact that chalcone synthase and STS compete for the same substrate (Jeandet et al., 1995; Vannozzi et al., 2012), the measured increase in stilbene accumulation in the exposed, compared to the shaded, berries may have depended on a combination of increased STS expression and higher substrate availability, provided by a possible inhibition of anthocyanin biosynthesis.

The differences in metabolic coordination between the exposed and shaded clusters in phenylpropanoid metabolism are intriguing. Exposing clusters to high SI generated a strong negative association between the precursor of the polyphenol pathway, phenylalanine, and the anthocyanins, which was not evident in the shaded clusters. In addition, in the exposed clusters, anthocyanins were more strongly correlated with narin-chalc-glu, but less with flavonols, and the correlation between flavonols and stilbenes shifted from highly negative (shaded) to partially positive (exposed). Anthocyanin accumulation in grape-tissue culture was recently shown to be decoupled from phenylalanine under conditions in which biosynthesis-related gene expression was down-regulated (Manela et al., 2015). As a result, the tissue accumulated higher levels of flavonols and stilbenes. It is possible that a similar phenomenon occurred in the fully exposed clusters, as biosynthetic gene expression may have been hampered under conditions that included elevated temperatures (Azuma et al., 2012). Taken together, these lines of evidence are in support of a repartitioning of carbon precursors of the polyphenol pathway from anthocyanin biosynthesis to that of stilbenes and flavonols. This hypothesis should be confirmed in future studies by implementing stable isotopes based flux analysis in detached berries.

Our results revealed that shifting the intensity and direction of solar irradiance (SI) significantly modulated the spatial patterns of the accumulation of organic and amino acids, the main sugars glucose and fructose, and the majority of skin phenylpropanoid metabolites, across the grape-cluster, while hydroxy-cinnamates were not affected. In addition, filtering the irradiance intensity significantly affected the levels of 24 metabolites across the spatial locations, including organic and amino acids, flavonols, flavan-3-ols, anthocyanins and stilbenes. The within-cluster spatial heterogeneity was characterized by large variations in the flavonol levels, found to be significantly affected by sunlight exposure. Overall, the effect of SI conditions on skin phenylpropanoids was comprehensive, while it was found to be more specific in the case of pulp primary metabolites. Together, these findings suggest that sunlight plays a major role in shaping the spatial accumulation of quality-related compounds within a single grape cluster.

The calculated fold changes in metabolite accumulation, across the spatial dataset presented here (Supplementary Table 3), summarizes the compound plasticity of 70 primary and secondary metabolites to light and temperature perturbations, revealing the potential modulating effect of sunlight regulation on fruit composition. Considering that a single layer of grapevine leaves absorbs at least 60–70% of the visible wavelengths (Schultz, 1996), the gradient of SI intensities and metabolite fold change values found in this study may represent, yet possibly underestimate, the range found within a single, non-defoliated, commercial vine.

## Conclusions

**Figure 8:**
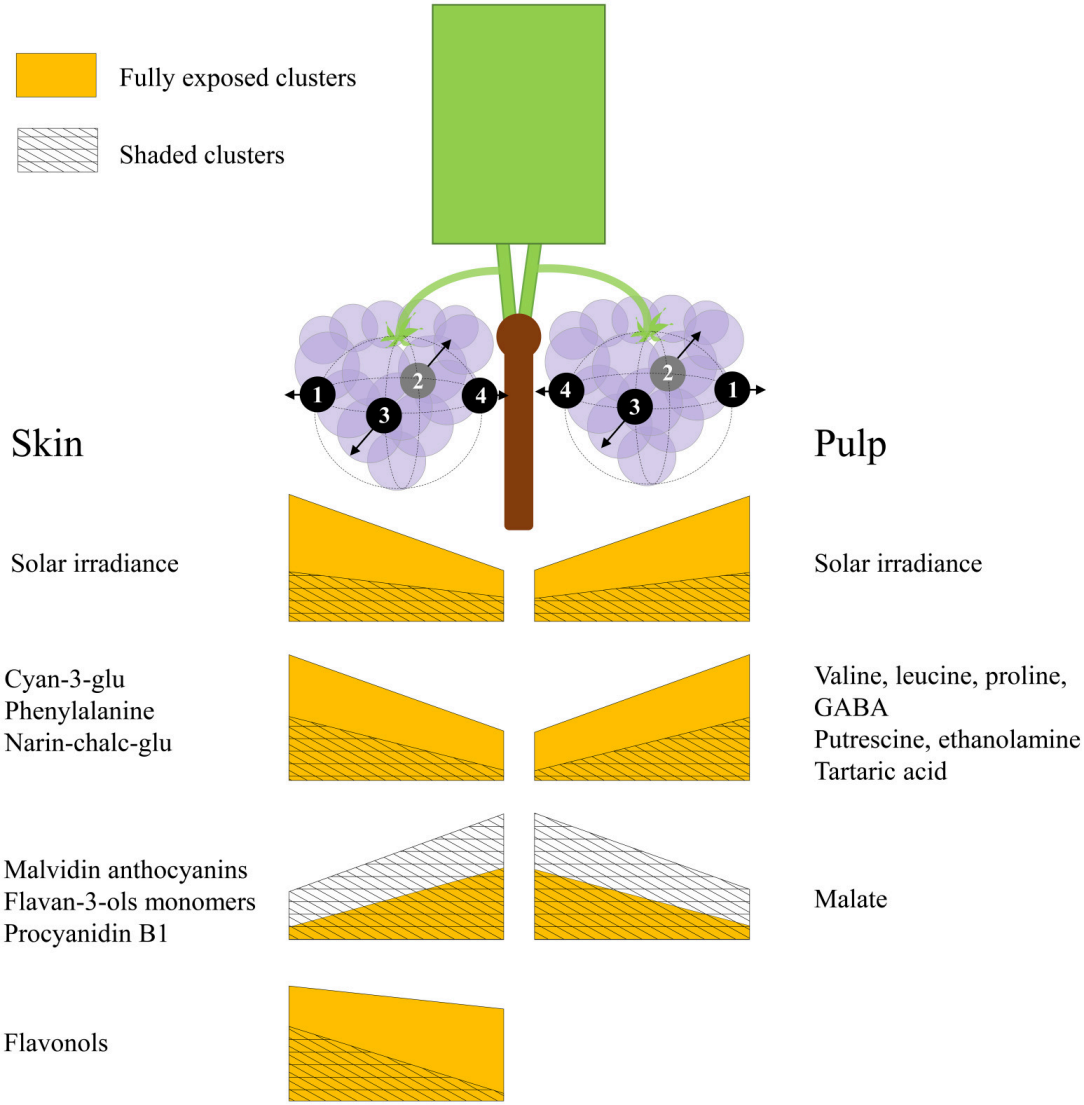
**An illustration summarizing the main findings of the study**. Both sides of the canopy refer to the same cluster orientations (mirror image), the left side displays the results obtained for skin phenylpropanoids, and the right side for pulp primary metabolites. The trapezoid heights represent metabolite abundance and its pattern of change across the cluster, from internal to external orientations, corresponding with daily incoming SI. Yellow trapezoids represent fully exposed clusters, while mesh trapezoids represent 60% shaded clusters.

Grape berries' acclimation to their surrounding environment involves local metabolic shifts, which affect their chemical composition and quality at harvest. Sunlight exposure triggers a complex response through both irradiance-mediated signaling and accumulated heat. Here, we show that this metabolic acclimation to sunlight drives the spatial variability of chemical composition between berries on a single cluster, and that it involves the interaction and modulation of partitioning between several biochemical processes in both pulp and skin. These include the accumulation of pyruvate and 2-oxoglutarate-derived nitrogenous compounds, at the expense of malate, fumarate and aspartate in the pulp, and the accumulation of phenylalanine, narin-chalc-glu, cyan-3-glu and the flavonols, accompanied with a decrease in flavan-3-ols, hydroxy-cinnamates and malvidin anthocyanins, in the skin. Figure 8 illustrates the main findings of the study.

This study characterized, yet did not isolate, the accompanying climatic components, such as temperature, that are known to influence fruit metabolic processes. Therefore, our findings may apply to a defined climate, namely warm and arid to semi-arid regions. The existing knowledge gap regarding the intriguing interaction between (SI) and berry temperature, as well as the contribution of temperature-driven processes to the overall fruit metabolic response, may hinder the extrapolation of our findings to systems experiencing different climatic conditions.

At the practical level, (SI) is the most easily and readily controlled climatic factor. In the context of climate change, these results will aid in designing a knowledge-based use of sunlight regulation as tool to improve grape composition, under the conditions that are expected to prevail in an ever-expanding number of commercial vineyards worldwide.

## Author contributions

NR, NA, and AF conceived and planned the study. NR and NW applied the viticultural treatments, and collected the meteorological data and berry samples in the field. NR and NW analyzed the meteorological data and prepared the berry samples for extraction. NW processed and analyzed the thermal images, and NR performed the sample extraction and analysis using the UPLC-QTOF-MS and GC-MS devices. NR integrated the data and performed the data analysis. NR wrote the body of the paper with AF and NA. All authors reviewed and approved the manuscript.

## Funding

This work was partially funded by the Koshland Foundation for the Support of Interdisciplinary Research in Combating Desertification, and the Frances and Elias Margolin Trust.

### Conflict of interest statement

The authors declare that the research was conducted in the absence of any commercial or financial relationships that could be construed as a potential conflict of interest.

## Abbreviations

GABA: gamma-Aminobutyric acid
Cyan-3-glu: Cyanidin-3-O-glucoside
Pet-3-glu: Petunidin-3-O-glucoside
Peo-3-glu: Peonidin-3-O-glucoside
Mal-3-glu: Malvidin-3-O-glucoside
Delph-3-glu: Delphindin-3-O-glucoside
Delph-3-acet: Delphinidin-3-O-(6″-acetyl-glucoside)
Cyan-3-acet: Cyanidin-3-O-(6″-acetyl-glucoside)
Pet-3-acet: Petundin-3-O-(6″-acetyl-glucoside)
Mal-3-acet: Malvindin-3-O-(6″-acetyl-glucoside)
Peo-3-acet: Peonidin-3-O-(6″-acetyl-glucoside)
Delph-3-coum: Delphinidin-3-O-(6″-p-coumaroyl-glucoside)
Mal-3-caffe: Malvidin-3-O-(6″-caffeoyl-glucoside)
Cyan-3-coum: Cyanidin-3-O-(6″-p-coumaroyl-glucoside)
Pet-3-coum: Petunidin-3-O-(6″-p-coumaroyl-glucoside)
Peo-3-coum: Peonidin-3-O-(6″-p-coumaroyl-glucoside)
Mal-3-coum: Malvidin-3-O-(6″-p-coumaroyl-glucoside)
Myr-3-glr: Myricetin-3-O-glucuronide
Rutin: Quercetin-3-O-rutinoside
Myr-3-glu: Myricetin-3-O-glucoside
Quer-3-glr: Quercetin-3-O-glucuronide
Quer-3-glu: Quercetin-3-O-glucoside
Kaemp-3-glr: Kaempferol-3-O-glucuronide
Kaemp-3-glu: Kaempferol-3-O-glucoside
Narin-chalc-glu: Naringenin-chalcone-4-O-glucoside
Hex.: Hexoside.
