# Supplementary material for: Sunlight Modulates Fruit Metabolic Profile and Shapes the Spatial Pattern of Compound Accumulation within the Grape Cluster
Source: Front Plant Sci. 2017 Feb 1;8:70. doi: 10.3389/fpls.2017.00070 (PMC5285383; doi:10.3389/fpls.2017.00070)
Supplement: Supplementary file 3 [file Image1.PDF]

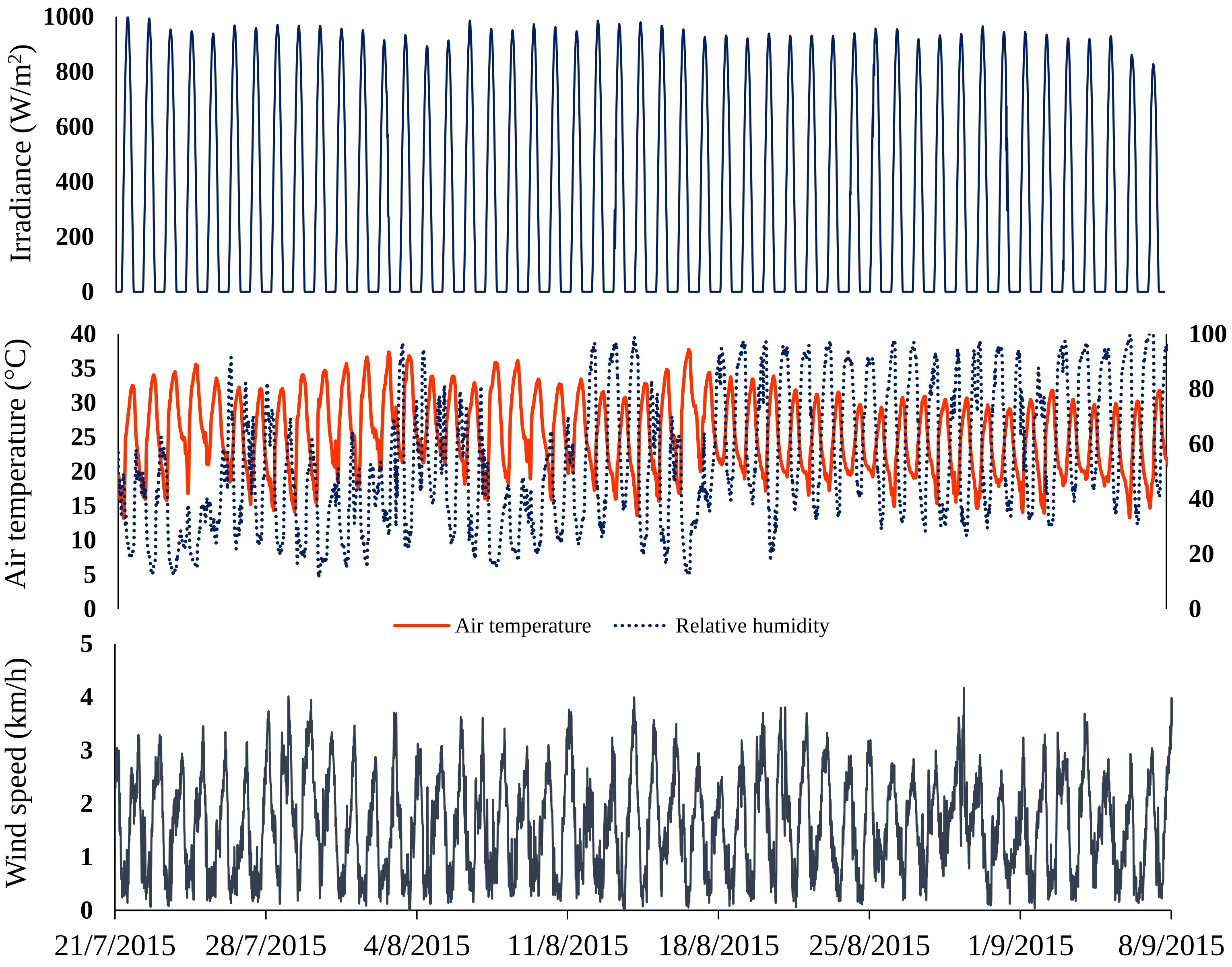


Supp. Fig. 1. Meteorological conditions at the experimental site: Wind speed, air temperature, relative humidity and irradiance, continuously measured above the canopy (vineyard scale) between veraison and sampling date, 2015.
