# Supplementary figures and images for: Sunlight Modulates Fruit Metabolic Profile and Shapes the Spatial Pattern of Compound Accumulation within the Grape Cluster
Source: Front Plant Sci. 2017 Feb 1;8:70. doi: 10.3389/fpls.2017.00070 (PMC5285383; doi:10.3389/fpls.2017.00070)

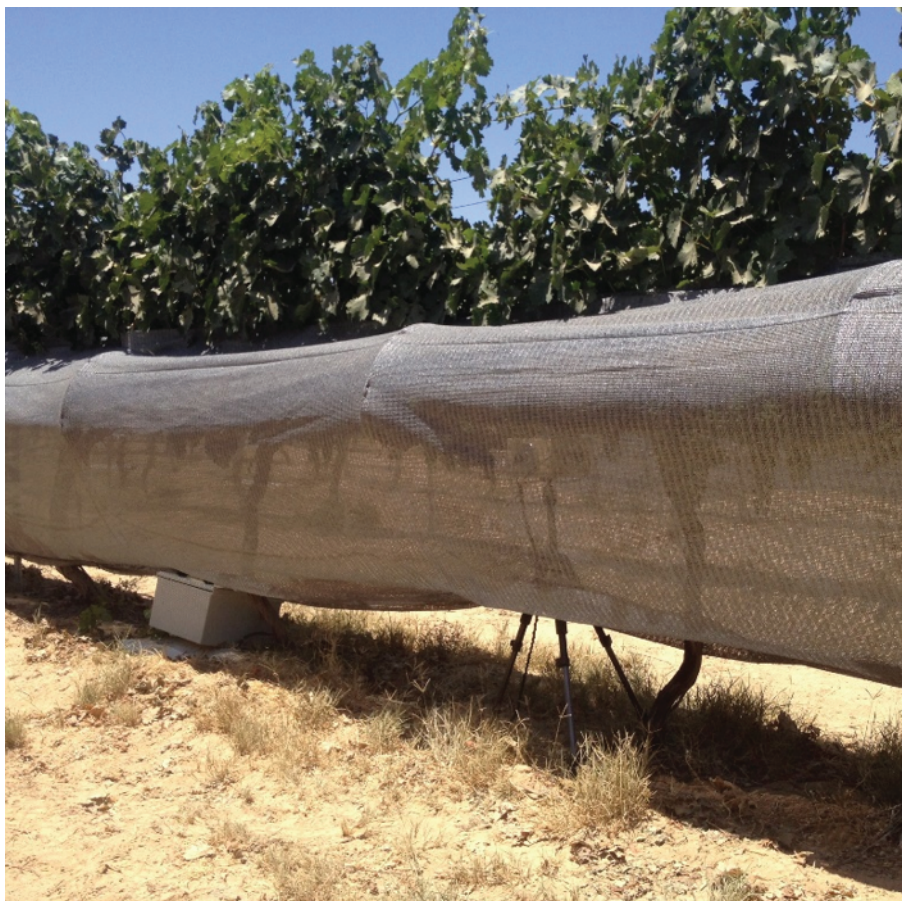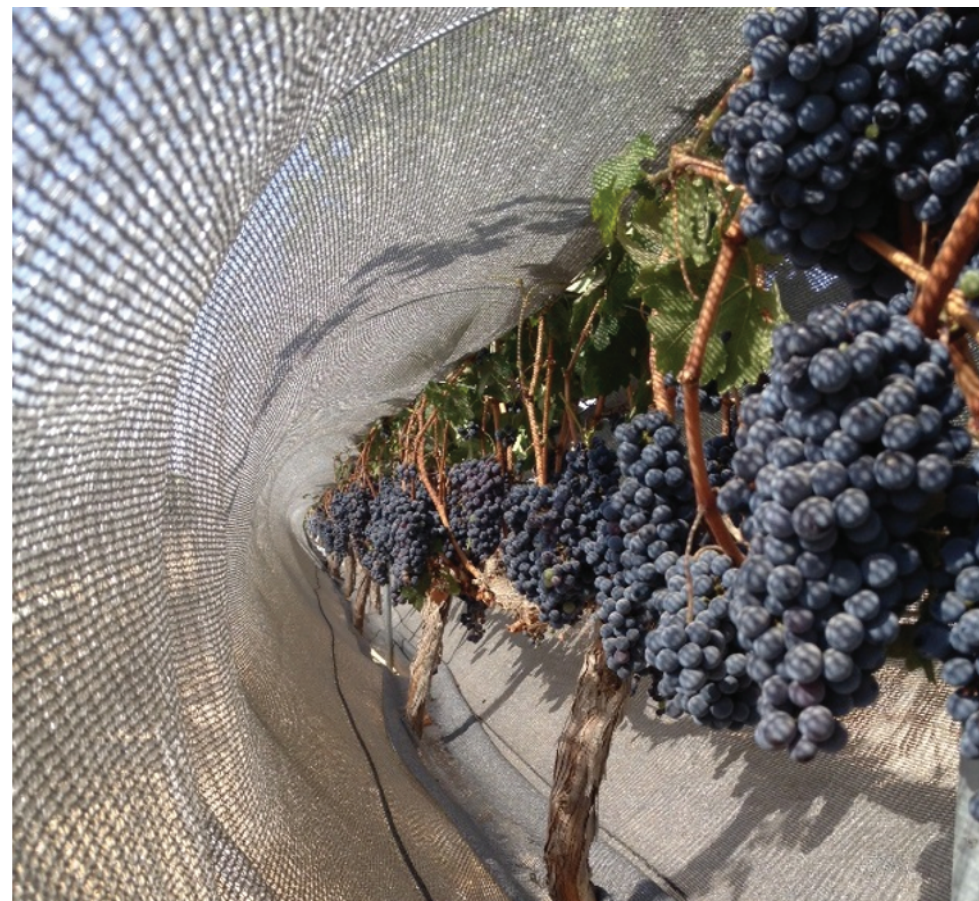

Supp. Fig. 2. Cluster zone shading net application design

Supplement: Supplementary file 4 [file Image2.PDF]

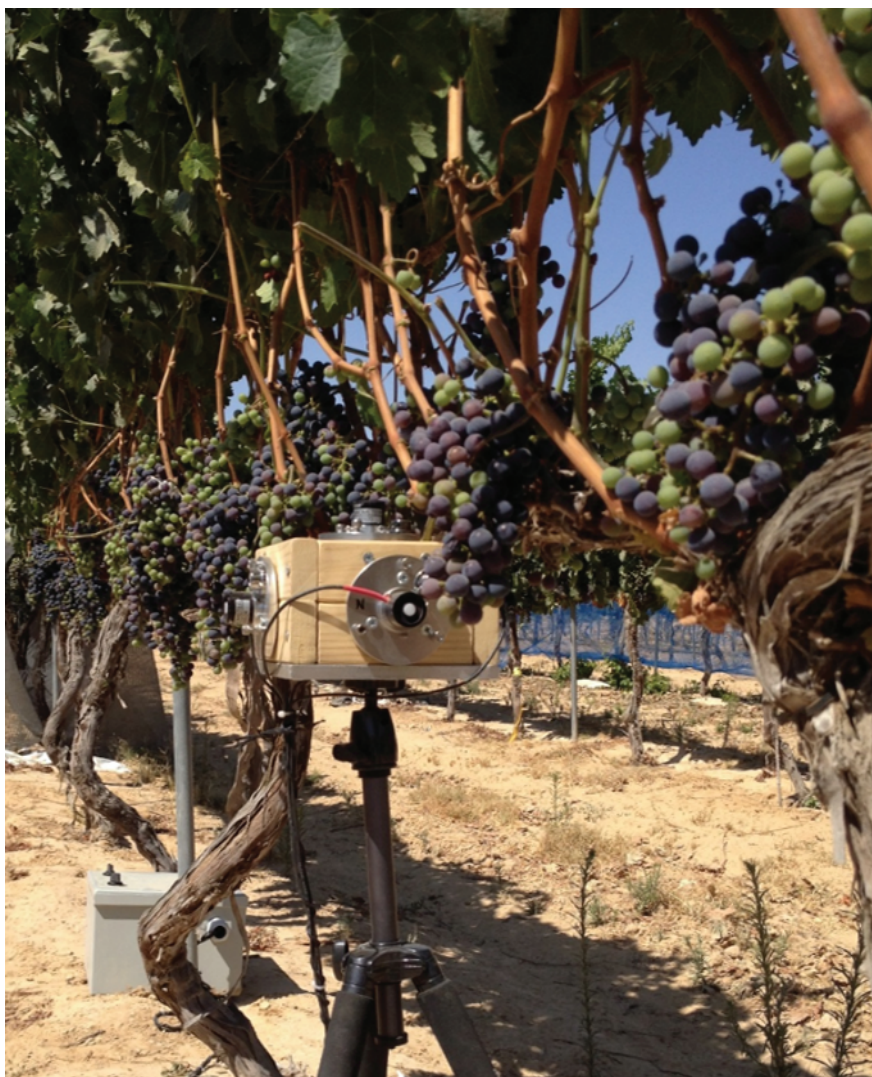

Supp. Fig. 3. Multi-pyranometer device for describing the irradiance regime at the cluster zone.

Supplement: Supplementary file 5 [file Image3.PDF]
