# Supplementary material for: Sunlight Modulates Fruit Metabolic Profile and Shapes the Spatial Pattern of Compound Accumulation within the Grape Cluster
Source: Front Plant Sci. 2017 Feb 1;8:70. doi: 10.3389/fpls.2017.00070 (PMC5285383; doi:10.3389/fpls.2017.00070)
Supplement: Supplementary file 6 [file Image4.PDF]

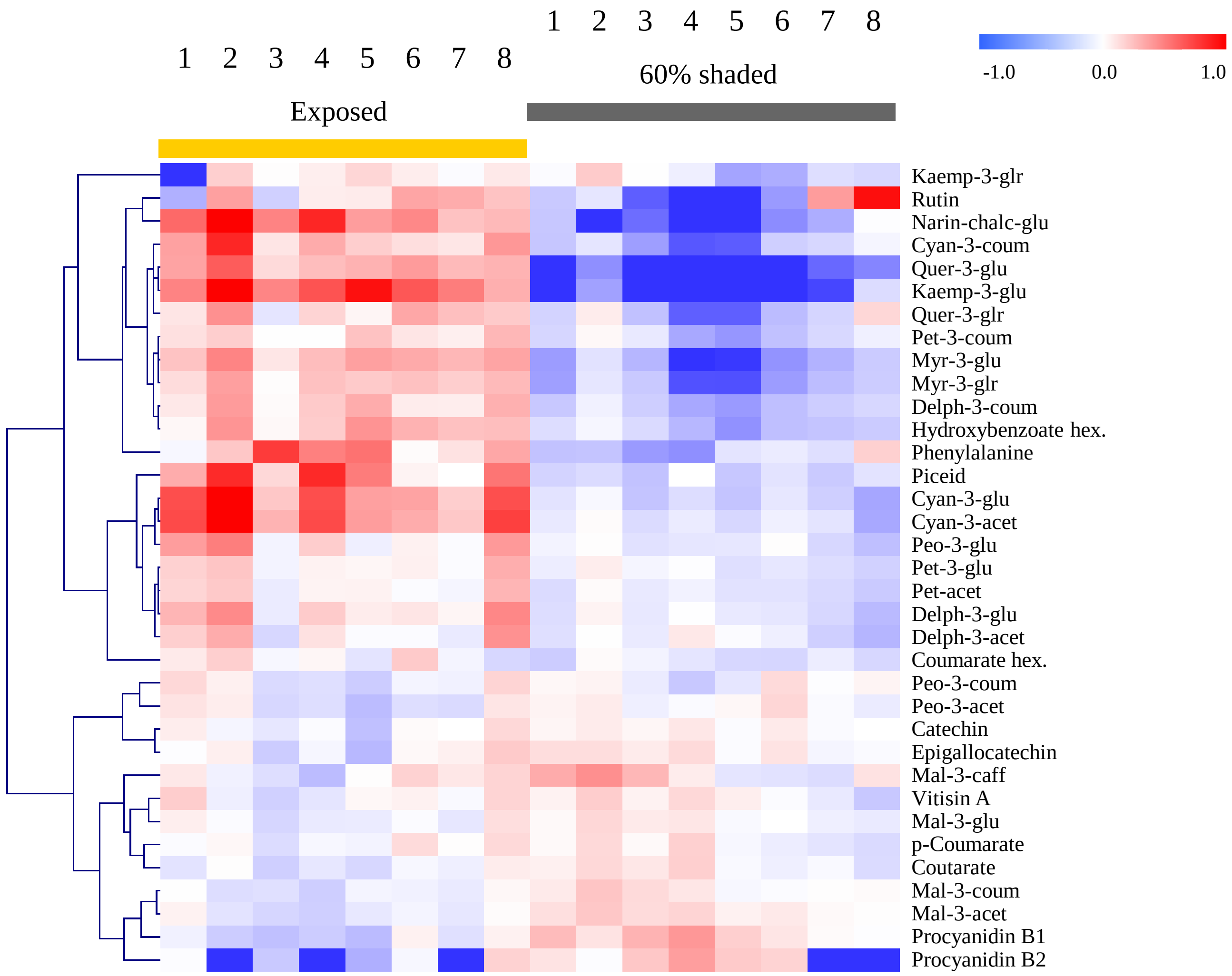

Supp. Fig. 4. Heatmap of grape skin phenylpropanoids across eight orientations (see fig.1) of clusters subjected to two sun exposure treatments: Fully exposed clusters (Exposed), and clusters shaded with 60% shading nets (60% shaded). The heatmap was generated with TMeV v4.9, using mean values of four biological replicates following normalization to the median of each metabolite based on all samples, and log2 transformation. Pearson correlation was used for hierarchical clustering of the metabolites.
