# Supplementary material for: Sunlight Modulates Fruit Metabolic Profile and Shapes the Spatial Pattern of Compound Accumulation within the Grape Cluster
Source: Front Plant Sci. 2017 Feb 1;8:70. doi: 10.3389/fpls.2017.00070 (PMC5285383; doi:10.3389/fpls.2017.00070)
Supplement: Supplementary file 7 [file Image5.PDF]

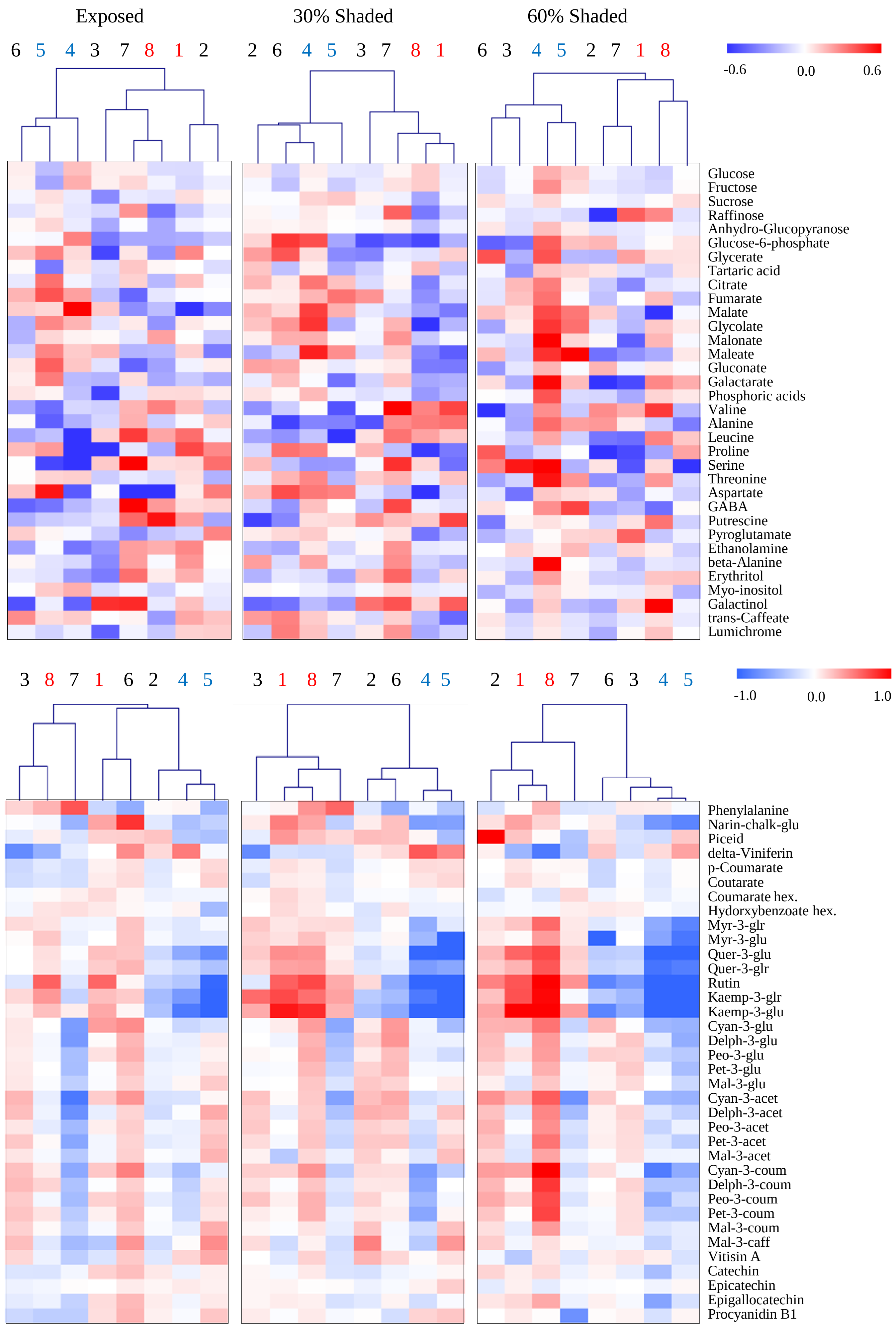

Supp. Fig. 5. Heatmap of grape pulp primary metabolites (A), and skin phenylpropanoids (B) across eight orientations (see fig.1) of clusters subjected to three sun exposure treatments: Fully exposed clusters (Exposed), clusters shaded with 30% shading nets (30% shaded) and clusters shaded with 60% shading nets (60% shaded). The heatmap was generated with TMeV v4.9, using mean values of four biological replicates. Values were normalized to the median of each specific cluster, following a log2 transformation. Pearson correlation was used for hierarchical clustering of cluster orientations done separately for each treatment.
