# Supplementary material for: Sunlight Modulates Fruit Metabolic Profile and Shapes the Spatial Pattern of Compound Accumulation within the Grape Cluster
Source: Front Plant Sci. 2017 Feb 1;8:70. doi: 10.3389/fpls.2017.00070 (PMC5285383; doi:10.3389/fpls.2017.00070)
Supplement: Supplementary file 8 [file Image6.PDF]

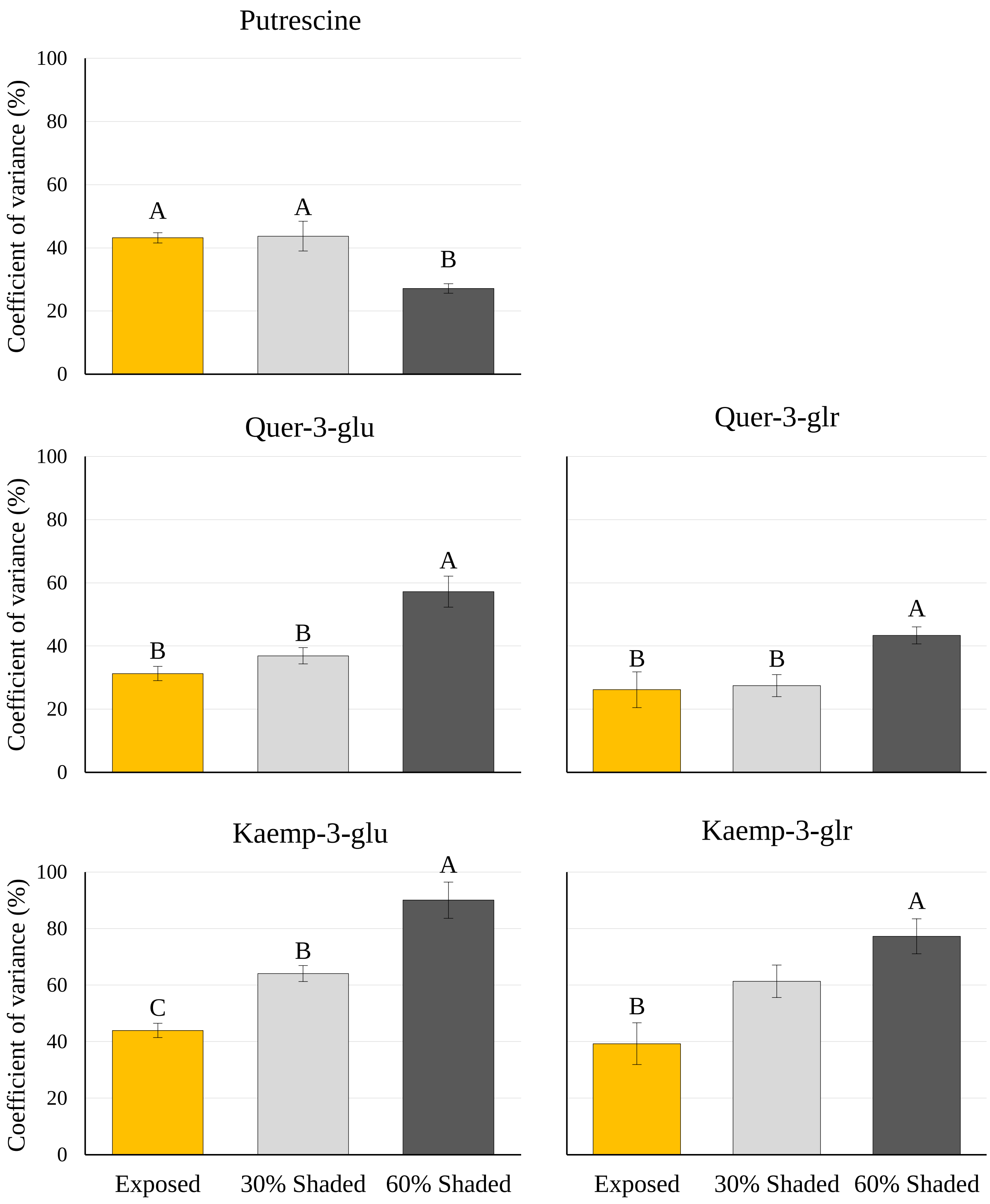

Supp. Fig. 6. Metabolites showing significant differences in within-vine coefficient of variance comparing three sun exposure treatments: Fully exposed clusters (Exposed), clusters shaded with 30% shading nets (30% shaded) and clusters shaded with 60% shading nets (60% shaded). Values are mean of four biological replicates. ANOVA and Tukey test were performed in R v3.3.1 using the “agricolae” package. Bars marked with different letters represent significantly different values ($\alpha<0.05$, n=4).
