# Supplementary material for: Sunlight Modulates Fruit Metabolic Profile and Shapes the Spatial Pattern of Compound Accumulation within the Grape Cluster
Source: Front Plant Sci. 2017 Feb 1;8:70. doi: 10.3389/fpls.2017.00070 (PMC5285383; doi:10.3389/fpls.2017.00070)
Supplement: Supplementary file 10 [file Image8.PDF]

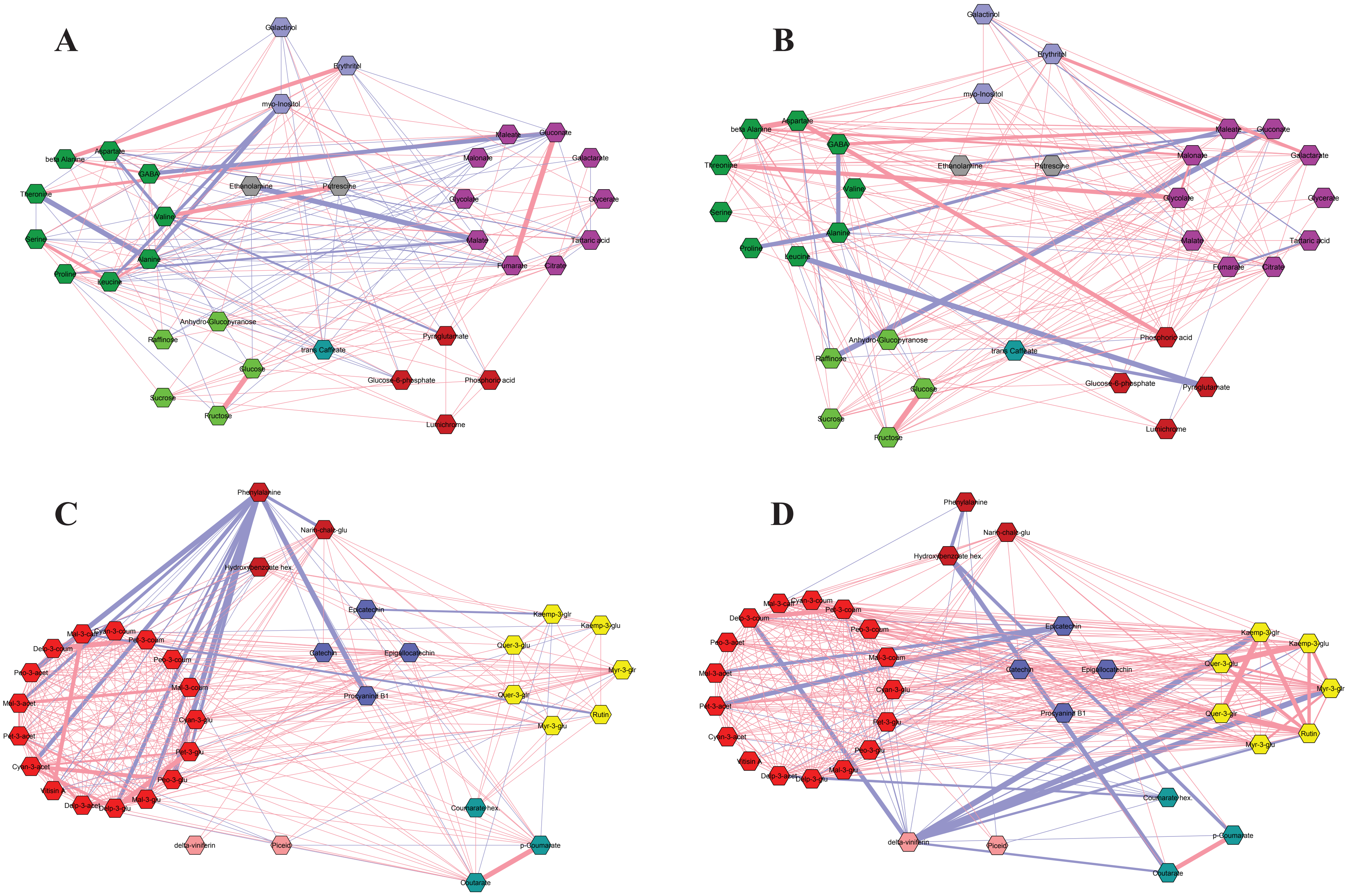


Supp Fig. 8. Correlation-based network of grape pulp primary metabolites and skin phenylpropanoids of clusters subjected to two sun exposure treatments: Fully exposed clusters (Exposed) and clusters shaded with 60% shading nets (60% shaded). A) primary metabolites of Exposed, B) primary metabolites of 60% Shaded, C) phenylpropanoids of Exposed and D) phenylpropanoids of 60% Shaded. The analysis was generated with Cytoscape V3.4.0, on mean values of four biological replicates.
